# Tau Positron Emission Tomography for Predicting Dementia in Individuals With Mild Cognitive Impairment

**DOI:** 10.1001/jamaneurol.2024.1612

**Published:** 2024-06-10

**Authors:** Colin Groot, Ruben Smith, Lyduine E. Collij, Sophie E. Mastenbroek, Erik Stomrud, Alexa Pichet Binette, Antoine Leuzy, Sebastian Palmqvist, Niklas Mattsson-Carlgren, Olof Strandberg, Hanna Cho, Chul Hyoung Lyoo, Giovanni B. Frisoni, Debora E. Peretti, Valentina Garibotto, Renaud La Joie, David N. Soleimani-Meigooni, Gil Rabinovici, Rik Ossenkoppele, Oskar Hansson

**Affiliations:** 1 Clinical Memory Research Unit, Department of Clinical Sciences in Malmö, Lund University, Lund, Sweden; 2 Alzheimer Center Amsterdam, Neurology, Vrije Universiteit Amsterdam, Amsterdam University Medical Center, Amsterdam, the Netherlands; 3 Department of Neurology, Skåne University Hospital, Lund University, Lund, Sweden; 4 Department of Radiology and Nuclear Medicine, Amsterdam University Medical Center, Vrije Universiteit Amsterdam, De Boelelaan, Amsterdam, the Netherlands; 5 Amsterdam Neuroscience, Brain Imaging, Amsterdam, the Netherlands; 6 Memory Clinic, Skåne University Hospital, Malmö, Sweden; 7 Wallenberg Center for Molecular Medicine, Lund University, Lund, Sweden; 8 Department of Neurology, Gangnam Severance Hospital, Yonsei University College of Medicine, Seoul, South Korea; 9 Memory Clinic, Department of Rehabilitation and Geriatrics, Geneva University and University Hospitals, Geneva, Switzerland; 10 Laboratory of Neuroimaging of Aging, University of Geneva, Geneva, Switzerland; 11 Laboratory of Neuroimaging and Innovative Molecular Tracers, Geneva University Neurocenter and Faculty of Medicine, University of Geneva, Geneva, Switzerland; 12 Division of Nuclear Medicine and Molecular Imaging, Geneva University Hospitals, Geneva, Switzerland; 13 Center for Biomedical Imaging, Geneva, Switzerland; 14 Department of Neurology, Memory and Aging Center, University of California, San Francisco; 15 Molecular Biophysics and Integrated Bioimaging Division, Lawrence Berkeley National Laboratory, Berkeley, California; 16 Department of Radiology and Biomedical Imaging, University of California, San Francisco; 17 Associate Editor, *JAMA Neurology*

## Abstract

**Importance:**

An accurate prognosis is especially pertinent in mild cognitive impairment (MCI), when individuals experience considerable uncertainty about future progression.

**Objective:**

To evaluate the prognostic value of tau positron emission tomography (PET) to predict clinical progression from MCI to dementia.

**Design, Setting, and Participants:**

This was a multicenter cohort study with external validation and a mean (SD) follow-up of 2.0 (1.1) years. Data were collected from centers in South Korea, Sweden, the US, and Switzerland from June 2014 to January 2024. Participant data were retrospectively collected and inclusion criteria were a baseline clinical diagnosis of MCI; longitudinal clinical follow-up; a Mini-Mental State Examination (MMSE) score greater than 22; and available tau PET, amyloid-β (Aβ) PET, and magnetic resonance imaging (MRI) scan less than 1 year from diagnosis. A total of 448 eligible individuals with MCI were included (331 in the discovery cohort and 117 in the validation cohort). None of these participants were excluded over the course of the study.

**Exposures:**

Tau PET, Aβ PET, and MRI.

**Main Outcomes and Measures:**

Positive results on tau PET (temporal meta–region of interest), Aβ PET (global; expressed in the standardized metric Centiloids), and MRI (Alzheimer disease [AD] signature region) was assessed using quantitative thresholds and visual reads. Clinical progression from MCI to all-cause dementia (regardless of suspected etiology) or to AD dementia (AD as suspected etiology) served as the primary outcomes. The primary analyses were receiver operating characteristics.

**Results:**

In the discovery cohort, the mean (SD) age was 70.9 (8.5) years, 191 (58%) were male, the mean (SD) MMSE score was 27.1 (1.9), and 110 individuals with MCI (33%) converted to dementia (71 to AD dementia). Only the model with tau PET predicted all-cause dementia (area under the receiver operating characteristic curve [AUC], 0.75; 95% CI, 0.70-0.80) better than a base model including age, sex, education, and MMSE score (AUC, 0.71; 95% CI, 0.65-0.77; *P* = .02), while the models assessing the other neuroimaging markers did not improve prediction. In the validation cohort, tau PET replicated in predicting all-cause dementia. Compared to the base model (AUC, 0.75; 95% CI, 0.69-0.82), prediction of AD dementia in the discovery cohort was significantly improved by including tau PET (AUC, 0.84; 95% CI, 0.79-0.89; *P* < .001), tau PET visual read (AUC, 0.83; 95% CI, 0.78-0.88; *P* = .001), and Aβ PET Centiloids (AUC, 0.83; 95% CI, 0.78-0.88; *P* = .03). In the validation cohort, only the tau PET and the tau PET visual reads replicated in predicting AD dementia.

**Conclusions and Relevance:**

In this study, tau-PET showed the best performance as a stand-alone marker to predict progression to dementia among individuals with MCI. This suggests that, for prognostic purposes in MCI, a tau PET scan may be the best currently available neuroimaging marker.

## Key Points

**Question:**

How well do visual reads and quantitative assessments of tau positron emission tomography (PET) predict clinical progression from mild cognitive impairment (MCI) to dementia compared to amyloid-β (Aβ) PET and magnetic resonance imaging (MRI)?

**Findings:**

In this cohort study, positivity on quantitative tau PET, but not Aβ PET or MRI, provided a better prediction of conversion from MCI to all-cause dementia when added to a base model including age, sex, education, and Mini-Mental State Examination score, while prediction of Alzheimer disease (AD) dementia was improved with quantitative tau PET as well as tau PET visual reads. The optimal set of neuroimaging biomarkers to predict all-cause and AD dementia included tau PET and MRI measures.

**Meaning:**

These findings suggest that quantitative tau PET and tau PET visual reads show the greatest promise as a stand-alone prognostic marker for clinical progression to dementia among individuals with MCI, outperforming Aβ PET and MRI.

## Teaser

This cohort study evaluates the use of tau positron emission tomography in estimating risk of progression to dementia in individuals with mild cognitive impairment.

## Introduction

Biomarkers to detect tau pathology, amyloid-β (Aβ) pathology, and neurodegeneration have caused a paradigm shift in the way Alzheimer disease (AD) is diagnosed, but they also show great promise as prognostic markers.^1,2,3,4^ An accurate, timely, and personalized prognosis helps individuals with AD to make future plans, reduce uncertainty regarding disease progression, and aid medical decision-making.^5^ Improvements to prognoses are especially pertinent in the mild cognitive impairment (MCI) stage, as individuals with MCI often experience considerable uncertainty and worry about future progression.^6^

Many biomarkers exist to capture AD pathology and neurodegeneration, but neuroimaging measures have shown the best performance in predicting future cognitive decline, especially in clinically more advanced stages of AD.^7,8^ Atrophy, as measured on structural magnetic resonance imaging (MRI), is one of the most widely implemented markers to predict progression to dementia in the clinic.^9^ However, MRI has low sensitivity for predicting subtle cognitive changes^10^ and is agnostic to the underlying etiology of clinical symptoms. Aβ positron emission tomography (PET) does capture one of the key pathological hallmarks of AD, resulting in an excellent sensitivity to detect AD.^11^ Aβ PET has also been shown to be associated with prediction of future cognitive impairment^12^ but studies showing these associations have generally required long follow-up times^13,14^ due to the considerable temporal delay between amyloidosis and clinical symptoms.^15^ Indeed, Aβ biomarker positivity and the onset of clinical symptoms may be up to 20 years apart,^16,17^ and many individuals with amyloid positivity never develop cognitive impairment during their lifetime.^4^ Since 2013, the second neuropathological hallmark of AD, tau protein depositions, can also be captured using PET.^18,19^ While not yet widely clinically implemented, tau PET shows accurate differentiation between individuals with AD and control individuals in research settings^18,20,21,22,23^ and has a close spatial and temporal association with clinical symptoms.^4,17^ Aside from AD, tau PET has also been shown to be elevated in 3R and 4R tauopathies, although differentiation against control individuals is not as clear as for AD.^24,25^ Multiple studies have also shown that tau PET signal in the neocortex is highly predictive of clinical progression in cognitively unimpaired individuals.^26,27^

We aim to examine the prognostic performance of tau PET in predicting clinical progression to dementia in individuals with MCI and to compare tau PET to neuroimaging markers that are already widely used in the clinical setting (ie, Aβ PET and MRI). Our analyses are tailored to maximize applicability in the clinic, and, therefore, where previous investigations have focused on selective cohorts, the current study includes a sample of individuals with MCI regardless of clinical presentation or biomarker status. For the same purpose, we will assess progression to any type of dementia (regardless of suspected etiology determined at dementia diagnosis) in addition to assessing AD dementia (ie, AD as suspected etiology at dementia diagnosis) specifically. To further maximize the potential clinical utility of our results, we will assess both dichotomized quantitative measures and visual reads, which are the most often implemented methods to score MRI and PET scans in the clinic. We hypothesize that tau PET, as assessed quantitatively or with visual reads, will show the greatest performance in predicting progression in individuals with MCI, and will outperform Aβ PET and MRI for prediction of both all-cause and AD dementia.

## Methods

### Standard Protocol Approvals, Registrations, and Patient Consents

All participants gave written informed consent. Ethical approval was given by ethical committees at each of the sites, and this study followed the Strengthening the Reporting of Observational Studies in Epidemiology (STROBE) reporting guideline.

### Participants

For the discovery cohort, we selected participants from a multicenter collaboration,^8,26,27,28,29,30,31^ including individuals from the Memory Disorder Clinic of Gangnam Severance Hospital, Seoul, South Korea; the Swedish BioFINDER-1 and BioFINDER-2 studies at Lund University, Lund, Sweden; the University of California, San Francisco, US; and Geneva University Hospitals Memory Clinics, Switzerland. The replication cohort was selected from the Alzheimer’s Disease Neuroimaging Initiative. Data were collected from June 1, 2014, to January 15, 2024. We only included participants with a baseline clinical diagnosis of MCI (see eAppendix 1 in Supplement 1 for criteria used across cohorts). Additional inclusion criteria were 1 or more clinical follow-up; Mini-Mental State Examination (MMSE) score greater than 22; and tau PET, Aβ PET, and MRI results available less than 1 year from the MCI diagnosis. The mean (SD) follow-up duration was 1.9 (1.0) years in the discovery cohort and 2.2 (1.2) years in the validation cohort. Individuals were classified as having stable MCI when they did not experience clinical progression to dementia over the duration of follow-up. Individuals who developed dementia were classified as having progressed, and we made an additional distinction between individuals who developed any type of dementia (regardless of suspected etiology—ie, all-cause dementia) and those who developed AD dementia. Race and ethnicity data were not collected per study protocol.

### Neuroimaging Processing

Neuroimaging acquisition methods for each cohort are outlined in eAppendix 2 in Supplement 1. All neuroimaging data were processed using previously described standard image processing pipelines,^8,26,27,28,29,30,31^ and processing was performed centrally at Lund University to ensure maximal possible harmonization of data (eAppendix 2 in Supplement 1). Tau PET was assessed within a temporal meta–region of interest (meta-ROI),^32^ global Aβ PET levels were assessed using the Centiloid scale,^33^ and cortical thickness was assessed in an AD signature region.^34^ Detailed neuroimaging processing methodology is outlined in eAppendix 2 in Supplement 1, as well as results using alternative ROIs for tau PET and MRI.

Because clinical assessments of neuroimaging markers most often rely on negative vs positive scores, we determined thresholds for positivity for all quantitative neuroimaging markers using the Youden index (ie, the highest combined value of sensitivity and specificity; *R* package cutpointr [R Foundation]) to maximize the distinction between those who progressed from stable MCI, and used these dichotomized scores in our analyses (eTable 1 in Supplement 1).

### Visual Reads

Detailed visual read methods are outlined in eAppendix 2 in Supplement 1. Briefly, accredited raters visually assessed neocortical tau PET (R.S. or C.G.), global Aβ PET (L.E.C., V.G., or C.G.), and medial temporal atrophy (MTA) on MRI (S.E.M., G.B.F., or C.G.). Alternative visual read methods with their corresponding results are outlined in eAppendix 2 and eFigures 2 and 3 in Supplement 1. For each modality, we randomly selected 30 cases, which were scored by 2 accredited assessors. Cohen κ was then used to determine the interrater agreement, which ranged from moderate (κ, 0.68; 95% CI, 0.40-0.97 for MTA scores) to near perfect (0.93; 95% CI, 0.79-1.00 for neocortical tau PET) (eTable 2 in Supplement 1).

### Statistical Analyses

Statistical analyses were performed in R version 4.2.2 (R Foundation). Receiver operating characteristic analyses were used to test the performance of the dichotomized, quantitative scores, and visual read scores to distinguish stable MCI from progression. We tested a model including only demographic characteristics (age, sex, education, and MMSE score) and covariates (cohort and follow-up time), and then assessed the additional benefit of each neuroimaging marker by individually adding it to this base model. We tested whether area under the receiver operating characteristic curve (AUC) statistics were significantly improved in the models including the neuroimaging markers compared to the base model and compared the AUC between neuroimaging models using the DeLong method.^35^ In addition, we performed Cox proportional hazard models to assess the risk of progression associated with each neuroimaging marker using the same set of predictors as for the receiver operating characteristic analyses. We also determined the added risk associated with having multiple positive biomarkers by computing a combined AT(N) variable (A = Aβ PET, T = tau PET, and N = MRI) using the quantitative scores. To determine the most parsimonious model to predict progression to dementia, we performed least absolute shrinkage and selection operator (LASSO) logistic regression, which accounts for interdependency of predictors. In the LASSO logistic regression, demographic characteristics (age, sex, education, and MMSE score) and covariates (cohort and follow-up time) were fixed variables.

## Results

### Demographic and Clinical Characteristics

The discovery cohort consisted of 331 individuals with a diagnosis of MCI at baseline. The mean (SD) age was 70.9 (8.5) years; 191 (58%) were male and 140 (42%) were female. A total of 165 individuals (52%) had an *APOEε4* allele, the mean (SD) MMSE score was 27.1 (1.9), and the mean (SD) clinical follow-up duration was 1.9 (1.0) years. A total of 110 individuals with MCI (33%) converted to dementia during follow-up (Table). Among them, 71 received a clinical diagnosis of AD dementia (65% of those who progressed) and the others (39 [35% of those who progressed]; grouped into the other dementia other group) were diagnosed with dementia other than AD or had an unknown etiology. This group consisted of frontotemporal dementia (n = 10), normal pressure hydrocephalus (n = 1), Parkinson disease dementia (n = 1), progressive supranuclear palsy (n = 2), vascular dementia (n = 10), dementia with Lewy bodies (n = 3), and unknown etiology (n = 12). Differences in demographic and clinical characteristics between those with stable MCI, those who progressed to AD dementia, and those who progressed to other dementia are displayed in the Table. For all subsequent analyses, the other dementia and AD dementia groups were combined to form the all-cause dementia group, while those who progressed to AD dementia were also assessed separately. eTable 4 in Supplement 1 outlines all demographic and clinical characteristics of the validation cohort.

**Table.** Baseline Demographic and Clinical Characteristics of the Discovery Cohort

Characteristic	Total sample, mean (SD)	Stable MCI, mean (SD)	Progressed to all-cause dementia, mean (SD)	*P* value vs stable MCI^a^	Progressed to AD dementia, mean (SD)^b^	*P* value vs stable MCI^a^	Progressed to other dementia, mean (SD)^b^	*P* value vs stable MCI^a^
No.	331	221	110	NA	71	NA	39	NA
Age, y	70.9 (8.5)	70.3 (8.6)	72.0 (8.1)	.09	73.1 (7.9)	.02^c^	70.0 (8.0)	.83
Sex, No. (%)								
Male	191 (57.7)	125 (56.6)	66 (60.0)	.63	41 (57.7)	.97	25 (64.1)	.48
Female	140 (42.3)	96 (43.4)	44 (40.0)		30 (42.3)		14 (35.9)	
Education, y	12.9 (4.1)	12.8 (3.7)	13.0 (4.7)	.78	13.2 (5.1)	.57	12.6 (3.9)	.76
*APOEε4*, No. (%)	165 (51.7)	97 (46.2)	68 (62.4)	.01^c^	52 (74.3)	<.001^c^	16 (41.0)	.67
Study, No. (%)								
BioFINDER-1^8,26,27,28,29,30,31^	26 (7.9)	16 (7.2)	10 (9.1)	<.001^c^	10 (14.1)	.01^c^	0	.03
BioFINDER-2^8,26,27,28,29,30,31^	212 (64.0)	139 (62.9)	73 (66.4)		43 (60.6)		30 (76.9)	
Gangnam Severance Hospital, Seoul^8,26,27,28,29,30,31^	41 (12.4)	24 (10.9)	17 (15.5)		13 (18.3)		4 (10.3)	
UCSF^8,26,27,28,29,30,31^	16 (4.8)	8 (3.6)	8 (7.3)		4 (5.6)		4 (10.3)	
Geneva University Hospitals^8,26,27,28,29,30,31^	36 (10.9)	34 (15.4)	2 (1.8)		1 (1.4)		1 (2.6)	
MMSE score	27.1 (1.9)	27.4 (1.8)	26.6 (2.0)	<.001^c^	26.5 (2.0)	<.001^c^	26.8 (1.9)	.08
Follow-up time, y	1.9 (1.0)	2.0 (1.0)	1.8 (1.0)	.05	1.8 (0.9)	.17	1.7 (1.0)	.09
Temporal meta–ROI tau, SUVR	1.4 (0.4)	1.3 (0.3)	1.5 (0.4)	<.001^c^	1.7 (0.4)	<.001^c^	1.2 (0.1)	.01
Tau PET visual read positive, No. (%)	112 (33.8)	61 (27.6)	51 (46.4)	<.001^c^	47 (66.2)	<.001^c^	4 (10.3)	.04
Aβ PET, Centiloids	50.4 (51.1)	42.9 (49.1)	65.4 (51.8)	<.001^c^	89.9 (40.4)	<.001^c^	20.6 (38.7)	.01
Aβ PET visual read positive	190 (57.4)	114 (51.6)	76 (69.1)	<.001^c^	64 (90.1)	<.001^c^	12 (30.8)	.03
AD signature cortical thickness, mm	2.4 (0.1)	2.4 (0.1)	2.3 (0.1)	<.001^c^	2.3 (0.1)	<.001^c^	2.3 (0.1)	.01
Medial temporal atrophy visual read positive, No. (%)	76 (23.0)	37 (16.7)	39 (35.5)	<.001^c^	25 (35.2)	<.001^c^	14 (35.9)	.01

Abbreviations: Aβ, amyloid β; AD, Alzheimer disease; MCI, mild cognitive impairment; MMSE, Mini-Mental State Examination; NA, not applicable; PET, positron emission tomography; ROI, region of interest; SUVR, standardized uptake value ratio; UCSF, University of California San Francisco.

^a^ Differences in continuous variables between progressors and stable MCI were assessed using pairwise independent *t* tests and by Fisher exact tests for categorical variables.

^b^ Progressed to AD dementia and progressed to other dementia are subgroups of the progressed to all-cause dementia group. Other dementia included frontotemporal dementia (n = 10), normal pressure hydrocephalus (n = 1), Parkinson disease dementia (n = 1), progressive supranuclear palsy (n = 2), vascular dementia (n = 10), dementia with Lewy bodies (n = 3), and unknown etiology (n = 12).

^c^ False discovery rate–corrected significant differences at α = .05 between groups.

### Performance of Each Neuroimaging Marker in Differentiating Between Stable MCI and Progression to Dementia

#### Discovery

**Figure 1.**
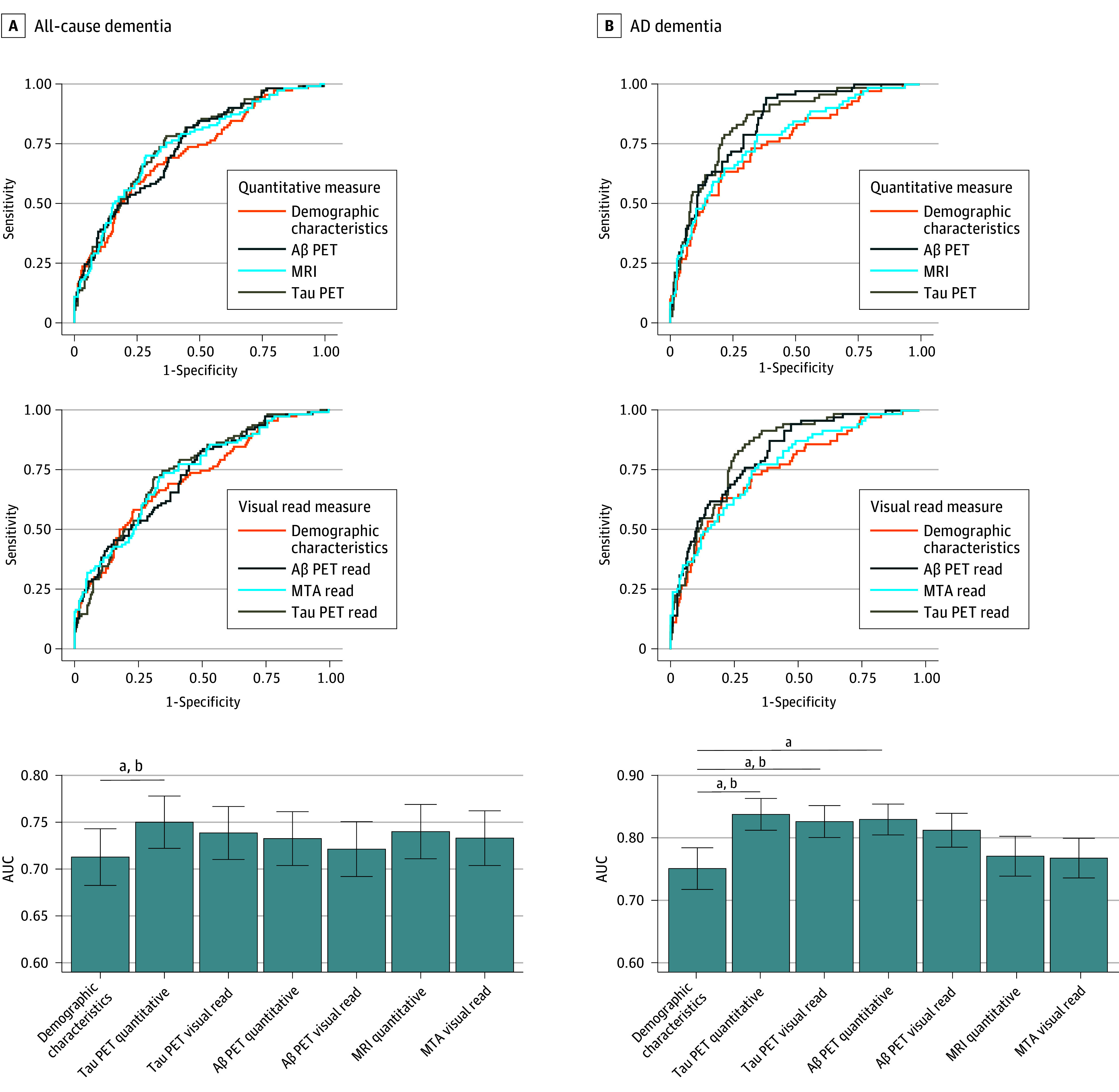
Area Under the Receiver Operating Characteristic Curve (AUC) Analyses to Distinguish Stable Mild Cognitive Impairment (MCI) From Progression to Dementia in the Discovery Cohort All models were corrected for age, sex, education, Mini-Mental State Examination score, cohort, and follow-up time. The scales for the AUC bar graphs are different between the analyses predicting all-cause dementia and Alzheimer disease (AD) dementia to best visualize the differences across neuroimaging measures. The error bars indicate the standard error. Aβ indicates amyloid β; MRI, magnetic resonance imaging; MTA, medial temporal atrophy; PET, positron emission tomography. ^a^Indicates that the AUC is significantly higher than the base model. Differences were assessed using the DeLong method. ^b^The differences in the discovery cohort indicate that the result was replicated in the validation cohort.

Only the model with temporal meta-ROI tau added predicted all-cause dementia better than a base model including age, sex, education, and MMSE score (AUC, 0.75; 95% CI, 0.70-0.80 vs AUC, 0.71; 95% CI, 0.65-0.77; *P* = .02). The models including tau PET visual reads (AUC, 0.74; 95% CI, 0.68-0.79; *P* = .06), Aβ PET (Centiloids: AUC, 0.73; 95% CI, 0.68-0.79; *P* = .32; visual read: AUC, 0.72; 95% CI, 0.66-0.78; *P* = .42), or MRI (AD signature thickness: AUC, 0.74; 95% CI, 0.68-0.80; *P* = .26; MTA visual read: AUC, 0.73; 95% CI, 0.68-0.79; *P* = .32) did not provide a better prediction for conversion to all-cause dementia than the base model (Figure 1A).

Compared to the base model (AUC, 0.75; 95% CI, 0.69-0.82), prediction of AD dementia was significantly improved by including temporal meta-ROI tau (AUC, 0.84; 95% CI, 0.79-0.89; *P* < .001), tau PET visual read (AUC, 0.83; 95% CI, 0.78-0.88; *P* = .001), and Centiloids (AUC, 0.83; 95% CI, 0.78-0.88; *P* = .03). Models including MRI (AD signature: AUC, 0.76; 95% CI, 0.70-0.83; *P* = .33; MTA visual read: AUC, 0.77; 95% CI, 0.71-0.83; *P* = .36) or Aβ PET visual read (AUC, 0.81; 95% CI, 0.76-0.87; *P* = .08) did not have better performance than the base model (Figure 1B).

#### Validation

**Figure 2.**
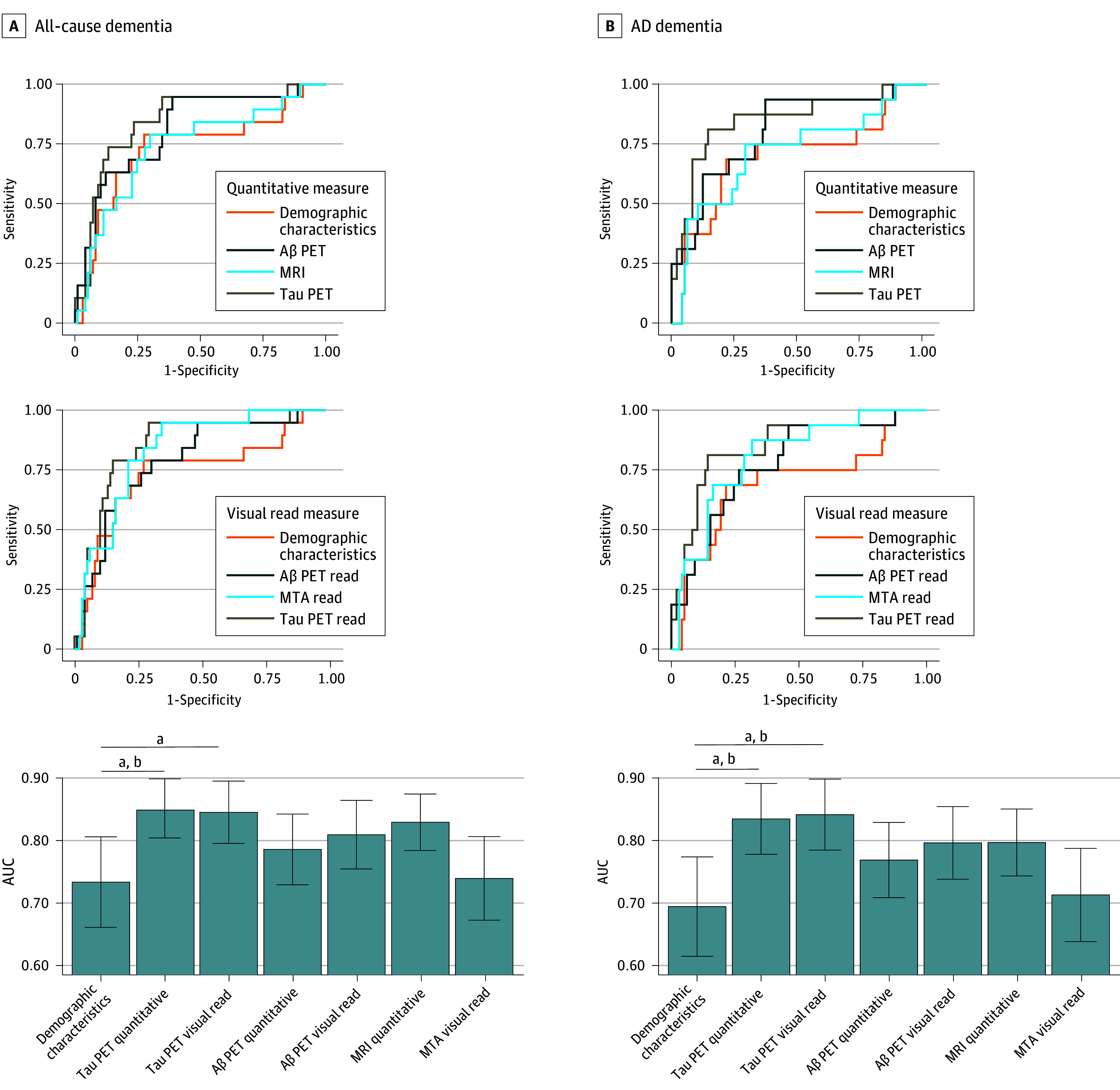
Validation of Area Under the Receiver Operating Characteristic Curve (AUC) Analyses to Distinguish Stable Mild Cognitive Impairment (MCI) From Progression to Dementia All models were corrected for age, sex, education, Mini-Mental State Examination score, and follow-up time. The scales for the AUC bar graphs are different between the analyses predicting all-cause dementia and Alzheimer disease (AD) dementia to best visualize the differences across neuroimaging measures. The error bars indicate the standard error. Aβ indicates amyloid β; MRI, magnetic resonance imaging; MTA, medial temporal atrophy; PET, positron emission tomography. ^a^Indicates that the AUC is significantly higher than the base model. Differences were assessed using the DeLong method. ^b^The differences in the discovery cohort indicate that the result was replicated in the validation cohort.

The increased performance of temporal meta-ROI tau to predict all-cause dementia over the base model (AUC, 0.85; 95% CI, 0.75-0.95 vs AUC, 0.73; 95% CI, 0.59-0.88; *P* = .04) was replicated in the validation cohort, while the model including tau PET visual read also performed better than the base model in the validation cohort (AUC, 0.85; 95% CI, 0.75-0.94; *P* = .04). The other neuroimaging markers again did not provide better predictive power than the base model (Figure 2A). For prediction of AD dementia, the increased performance over the base model (AUC, 0.70; 95% CI, 0.54-0.86) for the model including temporal meta-ROI tau (AUC, 0.85; 95% CI, 0.73-0.96; *P* = .04) and tau PET visual read (AUC, 0.85; 95% CI, 0.74-0.97; *P* = .03) were replicated in the validation cohort. However, the improved prediction of the model including Centiloids over the base model in the discovery cohort was not replicated in the validation cohort (AUC, 0.78; 95% CI, 0.65-0.90; *P* = .23) (Figure 2B).

Detailed statistics from all these models, including positive and negative predictive values, are presented in eTable 3 in Supplement 1. Line plots overlaying empirical and modeled receiver operating characteristic values indicated good calibration of our models (eFigure 1 in Supplement 1).

### Defining the Optimal Neuroimaging Model to Predict Progression to Dementia

#### Discovery

LASSO logistic regression analyses revealed that the optimal model for predicting all-cause dementia included a predictor for temporal meta-ROI tau (β, 0.89; SE, 0.28; *P* = .001), AD signature cortical thickness (β, 0.96; SE, 0.29; *P* = .001), and the MTA visual read (β, 0.71; SE, 0.30; *P* = .02). For predicting AD dementia, the model included temporal meta-ROI tau (β, 1.41; SE, 0.36; *P* < .001) and Centiloids (β, 1.87; SE, 0.52; *P* < .001).

#### Validation

Prediction for all-cause dementia included a predictor for temporal meta-ROI tau (β, 2.47; SE, 1.26; *P* = .049), tau PET visual read (β, 1.34; SE, 1.25; *P* = .28), and AD signature cortical thickness (β, 2.39; SE, 0.75; *P* = .001). For predicting AD dementia, the model included temporal meta-ROI tau (β, 3.47; SE, 0.93; *P* < .001) and the tau PET visual read (β, 2.36; SE, 0.76; *P* = .001). The Centiloid measure was not included in the optimal model in the validation cohort.

### Association Between Increased Risk of Dementia and Positivity on Each Neuroimaging Modality

**Figure 3.**
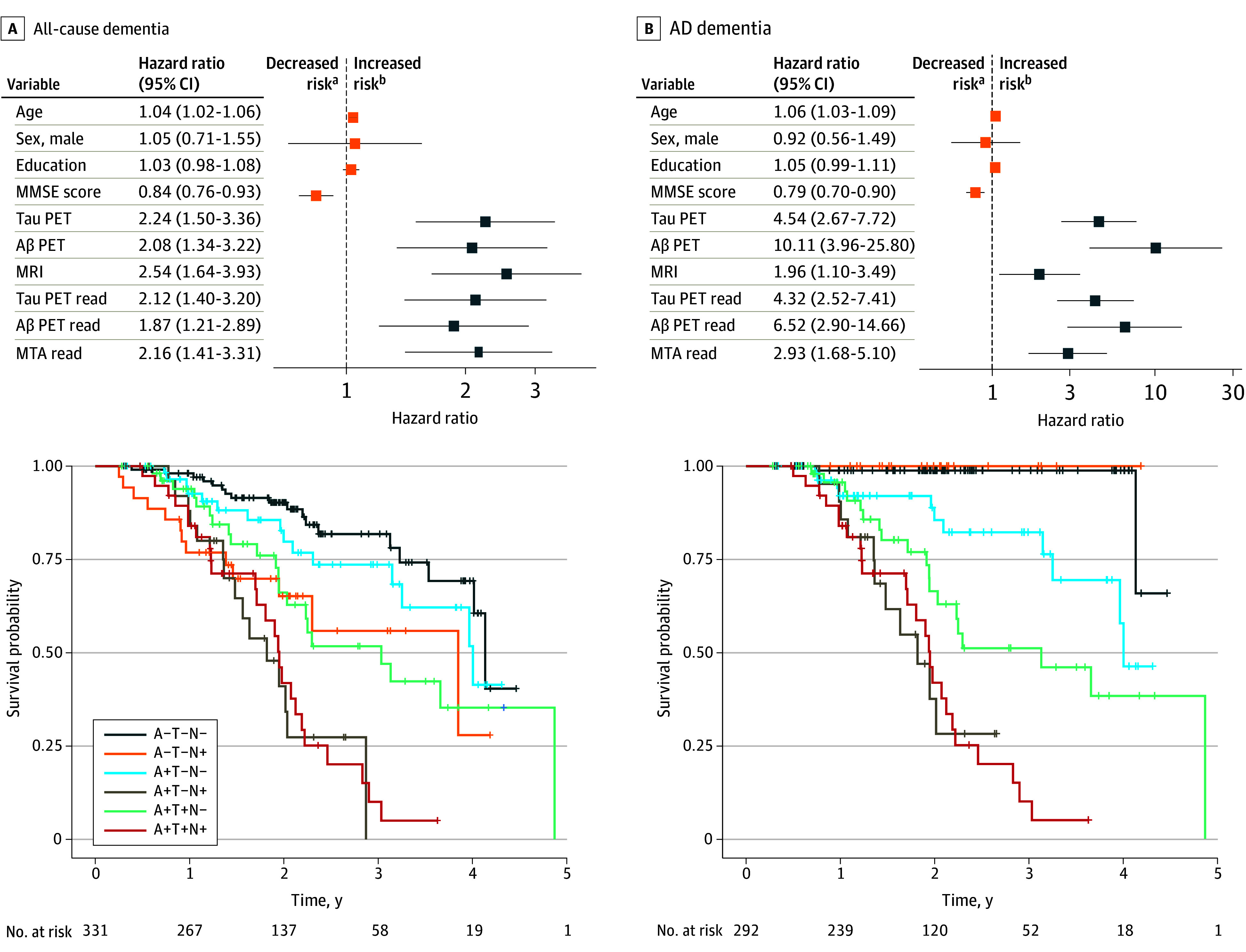
Association Between Risk of Clinical Progression From Mild Cognitive Impairment (MCI) to Dementia and Neuroimaging Biomarker Positivity in the Discovery Cohort Hazard ratios were obtained using Cox proportional hazard models using clinical follow-up as the time variable and controlling for cohort. The effects shown in orange were obtained from a model including only age, sex, education, and Mini-Mental State Examination (MMSE) score (ie, the base model). The effects shown in blue represent the hazard ratio from models including 1 neuroimaging measure added to the base model at a time. The Kaplan-Meier curves display hazard ratios associated with combinations of positive biomarkers, which were based on the quantitative markers. Groups: A−T−N− = 141, A+T−N− = 37, A−T+N- = 4, A−T−N+ = 58, A+T+N− = 71, A+T−N+ = 37, A−T+N+ = 5, A+T+N+ = 57. Curves were only displayed for groups of 5 or greater. Aβ indicates amyloid β; AD, Alzheimer disease; MRI, magnetic resonance imaging; MTA, medial temporal atrophy; PET, positron emission tomography. ^a^Decreased risk associated with higher scores (continuous variables; eg, Mini-Mental State Examination score) and for biomarker positivity (categorical variables; eg, tau PET). ^b^Increased risk associated with higher scores (continuous variables; eg, Mini-Mental State Examination score) and for biomarker positivity (categorical variables; eg, tau PET).

**Figure 4.**
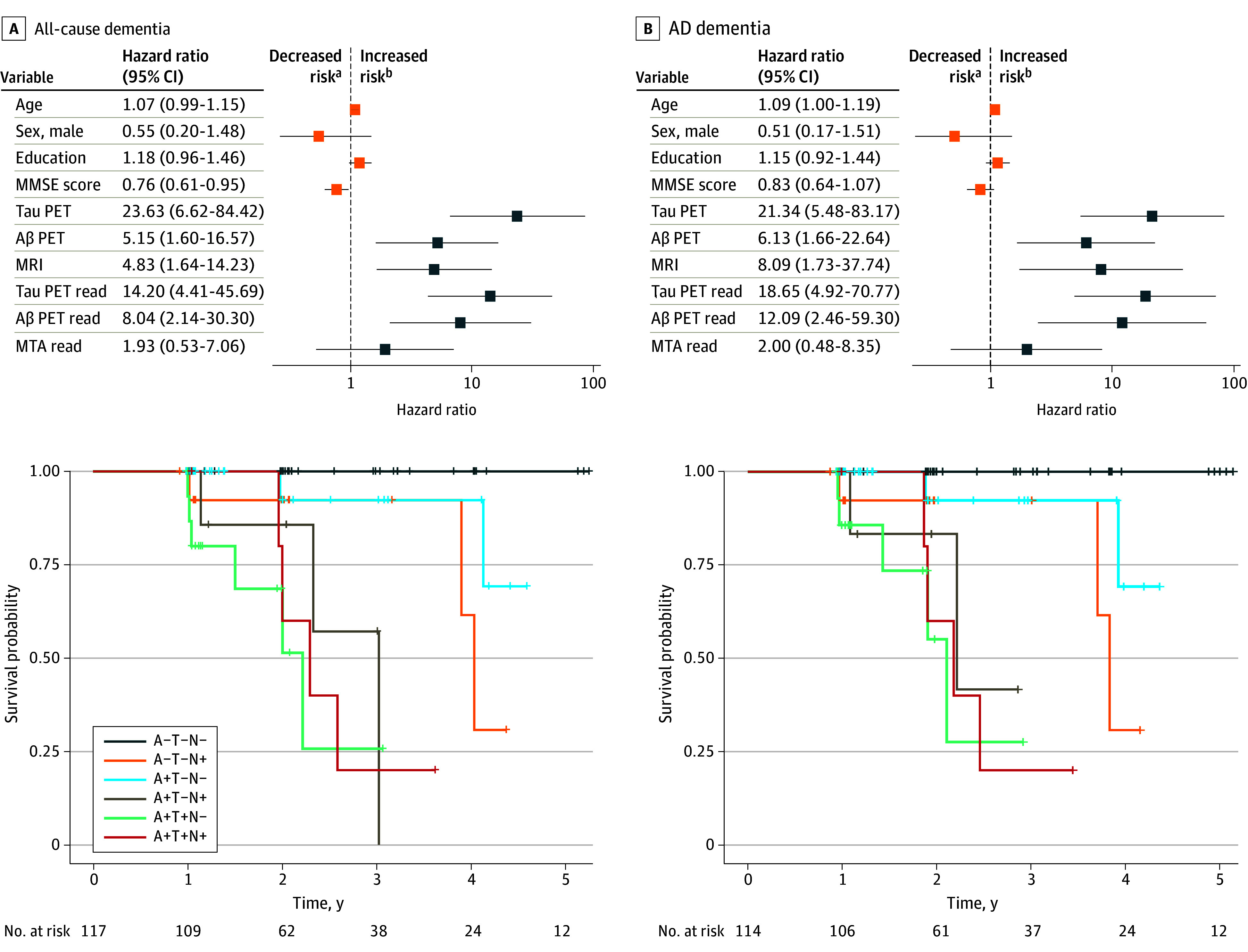
Validation of Cox Proportional Hazard Models Hazard ratios were obtained using Cox proportional hazard models using clinical follow-up as the time variable and controlling for cohort. The effects shown in orange were obtained from a model including only age, sex, education, and Mini-Mental State Examination (MMSE) score (ie, the base model). The effects shown in blue represent the hazard ratio from models including 1 neuroimaging measure added to the base model at a time. The Kaplan-Meier curves display hazard ratios associated with combinations of positive biomarkers, which were based on the quantitative markers. Groups: A−T−N− = 43, A+T−N− = 29, A−T+N− = 0, A−T−N+ = 14, A+T+N− = 16, A+T−N+ = 8, A−T+N+ = 1, A+T+N+ = 6. Curves were only displayed for groups of 5 or greater. Aβ indicates amyloid β; AD, Alzheimer disease; MRI, magnetic resonance imaging; MTA, medial temporal atrophy; PET, positron emission tomography. ^a^Decreased risk associated with higher scores (continuous variables; eg, MMSE score) and for biomarker positivity (categorical variables; eg, tau PET). ^b^Increased risk associated with higher scores (continuous variables; eg, MMSE score) and for biomarker positivity (categorical variables; eg, tau PET).

In both the discovery and validation cohorts, positivity on the quantified and visual read markers for tau PET, Aβ PET, and MRI conveyed an increased risk of converting to all-cause dementia and AD dementia, with prediction for AD dementia showing higher hazard risk ratios. The ATN biomarker profile groups revealed that having a combination of positive neuroimaging biomarkers conveyed a greater risk of progression to dementia than isolated positivity on 1 biomarker. The highest hazard ratios were associated with the A+T+N+ biomarker profile when predicting AD dementia (Figures 3 and 4).

### Sensitivity Analyses

Sensitivity analyses examining the effects of age (groups stratified at the mean; 70.9 for the discovery cohort and 72.7 for the validation cohort), sex (female vs male), and *APOEε4* (carriers vs noncarriers) on the prognostic performance of tau PET, Aβ PET, and MRI are presented in eFigures 4-6 in Supplement 1. In the discovery cohort, the performance of temporal meta-ROI and tau PET visual reads to predict all-cause dementia were better in younger individuals than in older individuals (eFigure 4 in Supplement 1) and all neuroimaging markers performed better in *APOEε4* noncarriers vs *APOEε4* carriers (eFigure 5 in Supplement 1) when predicting AD dementia. However, these results were not replicated in the validation cohort.

## Discussion

In this cohort study, we set out to examine the prognostic performance of tau PET, as assessed in the temporal meta-ROI and using visual reads, to predict dementia among individuals with MCI, and compare this performance to Aβ PET and MRI. We found that only temporal meta-ROI tau showed better performance to predict all-cause dementia than a base model of demographic characteristics, and this was replicated in our validation cohort. For prediction of AD dementia, only temporal meta-ROI tau and tau PET visual reads provided better performance than a base model that could be validated, while increased performance of Aβ PET over the base model in the discovery cohort could not be replicated in the validation cohort. Optimal models to predict all-cause and AD dementia only included tau PET and MRI measures in both the discovery and validation cohorts. Hazard ratios associated with biomarker profiles indicated that having a combination of positive biomarkers (eg, A+T+N+) conveyed a higher risk of progression than 1 positive biomarker in isolation. Taken together, our results suggest that tau PET had the best performance to predict clinical progression among individuals with MCI, performing better than Aβ PET and MRI. However, the most optimal prediction was achieved using a multimodal approach.

Contrary to MRI and Aβ PET, tau PET has not been adopted as a prognostic marker into routine clinical practice, even though tau pathology has been shown to be more strongly associated with clinical symptoms than atrophy and Aβ pathology.^4,17^ In line with these findings, previous examinations relating to the predictive effects of tau PET have highlighted an association between cognitive decline and tau PET,^8,36,37,38^ outperforming Aβ PET, MRI,^8^ and biofluid markers of phosphorylated tau.^7^ We expand on these earlier findings by showing that among individuals with MCI, tau PET was highly predictive for future progression to dementia and could be regarded the optimal stand-alone imaging marker in MCI. Progression to dementia is a highly relevant outcome to patients and caregivers, regardless of the etiology. This is why the current study, in contrast to previous examinations, included a cohort of individuals with MCI that was not preselected or stratified based on clinical presentation (eg, with vs without amnesia) or biomarker (eg, Aβ) status, and we assessed progression to any type of dementia. Another novel contribution to the existing literature is the assessment of visual reads. This is currently the only approved method of assessing tau PET scans, and visual reads of MRI and PET scans have historically been a mainstay in clinical assessments. We showed that tau PET visual read assessments had a comparable prognostic performance compared to dichotomized quantitative assessments. Visual interpretation of tau PET scans may enable a broader clinical application and could make tau PET more accessible to medical practitioners and, therefore, to patients. In line with a previous examination,^39^ our visual reads for tau PET had near perfect agreement between 2 raters, highlighting its reliability. Taken together with the potential of tau PET to accurately predict future progression, tau PET may contribute to diminish uncertainty about future progression in MCI, a stage when prognostic uncertainty is often considerable.^6^

MCI also constitutes a suitable stage to assess effects of disease-modifying treatments and recent clinical trials have focused on MCI and early AD.^40,41^ By highlighting the potential of tau PET as a tool to determine who will likely progress among an unselected cohort of individuals with MCI, our findings substantiate the utility of tau PET as a disease-monitoring tool in clinical trials. This is further substantiated when considering that we observed high predictive power even when our follow-up time was limited to approximately 2 years, which is in line with previous examinations that indicate a close temporal association between tau PET clinical symptoms.^42,43,44,45^ This close temporal association of tau PET with clinical progression also has clear beneficial implications for monitoring in treatment effects, as trials usually only have a follow-up of 18 months.^40,41,46^ Aside from a disease monitoring tool, tau PET has also been proposed as an inclusion tool into clinical trials.^40^ In this context, and especially with the advent of disease-modifying treatments, a biomarker with high sensitivity to detect pathological processes underlying clinical progression becomes imperative. Tau PET showed high specificity with a relatively minor reduction in sensitivity to detect progressors among our sample of individuals with MCI, resulting in significantly higher AUC statistics to detect conversion to dementia than a model including demographic characteristics only, and this result was replicated in an external validation cohort. None of the other neuroimaging markers showed a significant improvement in prediction of dementia over the base model that could be replicated in the external validation cohort. The replication of tau PET indicates that prediction of dementia was robust and reliable across cohorts, and also highlights the generalizability of the tau PET results. Reliability and generalizability of results are 2 crucial aspects that improve clinical utility of scientific results and corroborate the use of tau PET as a clinical trial inclusion tool and as a suitable marker to use when determining who will likely benefit most from treatment.

Also complementing earlier findings, we have outlined several sensitivity analyses assessing the performance of tau PET between age groups, sexes, and *APOEε4* status and found that tau PET performed better in younger than in older individuals and that all neuroimaging markers performed better in *APOEε4* noncarriers. These findings could not be replicated in our validation cohort. However, these results highlights that patient characteristics need to be considered when tailoring a prognosis in the clinic and in clinical trials, age and *APOEε4* status in particular.

### Strengths and Limitations

Strengths of the current study include replication of our results in an independent and external validation cohort, and the implementation of a large multicenter cohort of individuals with MCI who have longitudinal clinical follow-up and multimodal neuroimaging data available. Our study also has limitations, the first being our relatively short follow-up duration of approximately 2 years. This follow-up time does correspond to typical clinical trial durations and is also a representative follow-up time in the clinic but did not allow us to assess the long-term predictive power of our neuroimaging markers. Also, our sample consisted predominantly of highly educated individuals, highlighting the need for studies in more diverse samples. Furthermore, we selected often implemented and promising ROIs for tau PET and MRI to define biomarker positivity but alternative ROIs to define biomarker positivity for these measures exist. We provide insight into the predictive power of tau PET and MRI when using alternative ROIs in eFigures 2 and 3 in Supplement 1 but the results outlined in the main text might not be directly comparable to other studies using different ROIs. Additionally, there are inherent limitations associated with a multicenter design, such as data pooling, harmonization, and differing neuroimaging methodologies. These difficulties were partly addressed by central processing at Lund University by implementing the standardized Aβ PET measure of Centiloids, and eFigure 7 in Supplement 1 highlights that tau PET, Aβ PET, and MRI measures did not vary significantly between samples in the discovery cohort. However, issues with regard to heterogenous data are never completely overcome. Heterogeneity was also a feature of our other dementia group, which consisted of individuals with frontotemporal dementia, normal pressure hydrocephalus, Parkinson disease dementia, vascular dementia, progressive supranuclear palsy, dementia with Lewy bodies, or an unknown etiology. This might complicate the interpretation of the mechanisms underlying our findings with regard to the predictive effects of tau PET on conversion to all-cause dementia (where the other dementia group was included). A potential explanation for the performance of tau PET to predict conversion to all-cause dementia may be 3-fold: (1) a significant proportion of individuals with AD dementia (ie, 3R/4R tauopathy) may have been part of this group, and tau aggregation is closely related to neuronal dysfunction and cognitive decline; (2) some individuals with primary 3R and 4R tauopathies may have been detected by tau PET; and (3) possible comorbid tauopathy may have had a synergistic impact on cognitive decline. Taken together, the ability of tau PET to detect tauopathy related cognitive decline in a large proportion of individuals with MCI made it the most predictive marker for all-cause dementia in our analyses.

## Conclusions

In conclusion, with this study we show that tau-PET had superior predictive power over Aβ PET and MRI when predicting dementia. From a clinical and pragmatic perspective, knowing which neuroimaging modality to prescribe when aiming to estimate the chances of someone progressing or remaining stable within MCI clinical stage is imperative. This prognostic information is of vital importance to patients and caregivers, as recently highlighted in a published survey where “How quickly does my memory deteriorate?” was reported to be one of the most important questions for patients and caregivers.^6^ With tau PET being available in the US for diagnostic purposes and awaiting approval in other regions, the better prognostic performance of this marker over currently used Aβ PET and MRI measures has clinical relevance. Our findings constitute a next step toward the ultimate goal of accurate and individualized prognoses, which may reduce uncertainty and lower disease burden for individuals with MCI.

## Notes

Supplement 1. eAppendix 1. Diagnostic criteria for mild cognitive impairment across cohorts eAppendix 2. Detailed neuroimaging methods eTable 1. Thresholds for positivity across modalities eTable 2. Inter-rater agreement for tau-PET, amyloid-PET and MRI visual reads eTable 3. Receiver operating curve analysis assessing the predictive power of neuroimaging to detect progressors among individuals with MCI eTable 4. Baseline demographic and clinical characteristics of the validation cohort eFigure 1. Calibration plots for receiver operating characteristic analyses eFigure 2. Differences in tau-PET, Aβ-PET and MRI measures between individuals with stable MCI and progressors across alternative ROIs eFigure 3. Performance of all neuroimaging markers, including alternative ROIs, to detect progressors among individuals with MCI eFigure 4. ROC analyses to distinguish stable MCI from progressors, stratified according to age groups at the mean eFigure 5. ROC analyses to distinguish stable MCI from progressors, stratified according to APOEε4 genotype eFigure 6. ROC analyses to distinguish stable MCI from progressors, stratified according to sex eFigure 7. Data distribution of primary neuroimaging markers across cohorts in the discovery cohort

Supplement 2. Data sharing statement
