# Supplementary material for: Tau Positron Emission Tomography for Predicting Dementia in Individuals With Mild Cognitive Impairment
Source: JAMA Neurol. 2024 Jun 10;81(8):845–56. doi: 10.1001/jamaneurol.2024.1612 (PMC11165418; doi:10.1001/jamaneurol.2024.1612)
Supplement: Supplement 1. — eAppendix 1. Diagnostic criteria for mild cognitive impairment across cohorts eAppendix 2. Detailed neuroimaging methods eTable 1. Thresholds for positivity across modalities eTable 2. Inter-rater agreement for tau-PET, amyloid-PET and MRI visual reads eTable 3. Receiver operating curve analysis assessing the predictive power of neuroimaging to detect progressors among individuals with MCI eTable 4. Baseline demographic and clinical characteristics of the validation cohort eFigure 1. Calibration plots for receiver operating characteristic analyses eFigure 2. Differences in tau-PET, Aβ-PET and MRI measures between individuals with stable MCI and progressors across alternative ROIs eFigure 3. Performance of all neuroimaging markers, including alternative ROIs, to detect progressors among individuals with MCI eFigure 4. ROC analyses to distinguish stable MCI from progressors, stratified according to age groups at the mean eFigure 5. ROC analyses to distinguish stable MCI from progressors, stratified according to APOEε4 genotype eFigure 6. ROC analyses to distinguish stable MCI from progressors, stratified according to sex eFigure 7. Data distribution of primary neuroimaging markers across cohorts in the discovery cohort [file jamaneurol-e241612-s001.pdf]

Supplemental Online Content

Groot C, Smith R, Collij LE, et al. Tau positron emission tomography for predicting dementia in individuals with mild cognitive impairment. *JAMA Neurol*. Published online June 10, 2024. doi:10.1001/jamaneurol.2024.1612

eAppendix 1. Diagnostic criteria for mild cognitive impairment across cohorts
eAppendix 2. Detailed neuroimaging methods
eTable 1. Thresholds for positivity across modalities
eTable 2. Inter-rater agreement for tau-PET, amyloid-PET and MRI visual reads
eTable 3. Receiver operating curve analysis assessing the predictive power of neuroimaging to detect progressors among individuals with MCI
eTable 4. Baseline demographic and clinical characteristics of the validation cohort
eFigure 1. Calibration plots for receiver operating characteristic analyses
eFigure 2. Differences in tau-PET, Aβ-PET and MRI measures between individuals with stable MCI and progressors across alternative ROIs
eFigure 3. Performance of all neuroimaging markers, including alternative ROIs, to detect progressors among individuals with MCI
eFigure 4. ROC analyses to distinguish stable MCI from progressors, stratified according to age groups at the mean
eFigure 5. ROC analyses to distinguish stable MCI from progressors, stratified according to APOEε4 genotype
eFigure 6. ROC analyses to distinguish stable MCI from progressors, stratified according to sex
eFigure 7. Data distribution of primary neuroimaging markers across cohorts in the discovery cohort

This supplemental material has been provided by the authors to give readers additional information about their work.

© 2024 Groot C et al. *JAMA Neurology.*

eAppendix 1. Diagnostic criteria for mild cognitive impairment across cohorts

Discovery cohort:

BioFINDER 1 and 2 studies:

Mild cognitive impairment was determined based on established consensus criteria for mild neurocognitive disorder set out in the Diagnostic and Statistical Manual of Mental Disorders, Fifth Edition (DSM-5).

Gangnam Severance Hospital, Seoul:

MCI was diagnosed using the ICD-10-CM codes and includes F067. See https://www.aapc.com/codes/icd-10-codes/G31.84#:~:text=ICD%2D10%2DCM%20Code%20for,84 for more information.

University of California San Fransisco (UCSF):

At UCSF a clinical diagnosis of mild cognitive impairment was based on recommendations from the National Institute on Aging-Alzheimer's Association workgroups on diagnostic guidelines for Alzheimer's disease[6] and additionally had an Clinical Dementia Rating, (CDR) of 0.5.

Geneva University Hospitals Memory Clinic (GMC):

At GMC a clinical diagnosis of mild cognitive impairment was based on recommendations from the National Institute on Aging-Alzheimer's Association workgroups on diagnostic guidelines for Alzheimer's disease[6].

Validation cohort:

ADNI:

Criteria for MCI were based on the Petersen criteria[5]: 1) subjective memory complaints reported by themselves, or informant; 2) objective memory impairment below education-adjusted cut-off score on delayed recall of Story A of the WMS-R Logical Memory; 3) global CDR score of 0.5; and 4) general cognitive and functional performance sufficiently preserved such that a diagnosis of dementia could not be made.

© 2024 Groot C et al. *JAMA Neurology.*

eAppendix 2. Detailed neuroimaging methods

Acquisition

Tau-PET tracers used were [^{18}F]RO948 in BioFINDER-2 (n=116, 26%) and [^{18}F]flortaucipir/Tauvid in the other cohorts. Aβ-PET scans were obtained using [^{11}C]PiB at UCSF, using [^{18}F]florbetapir at a subset of ADNI sites, using [^{18}F]florbetaben in Seoul and the remaining ADNI sites, using [^{18}F]flutemetamol at BioFINDER-1 and BioFINDER-2, and using [^{18}F]florbetapir or [^{18}F]flutemetamol at Geneva University Hospitals Memory Clinic. Structural T1-weighted MRI was acquired on the following scanners: 3T-Discovery MR750 (GE Healthcare) in Seoul, 3T-Tim Trio (Siemens) or 3T-Prisma (Siemens) in BioFINDER-1 and BioFINDER-2, 3T-Tim Trio or 3T-Prisma (Siemens) at UCSF, 3T Magnetom Skyra, (Siemens) at Geneva University Hospitals, and a multitude of 1.5T and 3T scanners for ADNI (see: https://adni.loni.usc.edu/ for details).

Processing

All neuroimaging data were centrally processed at Lund University to ensure maximal possible harmonization of data. For tau-PET this processing includes resampling to obtain uniform image size (128 × 128 × 63 matrix) and voxel dimensions (2.0 × 2.0 × 2.0 mm), motion correction using AFNI's 3-dimensional volume registration, calculation of mean time, and rigid coregistration. Voxelwise standardized uptake value ratio (SUVR) images were created using the inferior cerebellar cortex as reference region[1-3]. FreeSurfer (v6.0, https://surfer.nmr.mgh.harvard.edu/) parcellation of the T1-weighted MRI scan in subject-space was used to extract mean regional SUVR values within Desikan-Killiany regions-of-interest (ROI). Amyloid-PET scans were converted into SUVR images using the cerebellum as the reference region and we applied a cortical mask to extract global Aβ-PET levels. We then applied tracer-specific conversion formulas to convert this global SUVR value into the Centiloid scale, which is a standard framework for the quantification of Aβ-PET scans across tracers and cohorts[4]. Cortical reconstruction of MRI images was performed with the FreeSurfer (v6.0) image analysis pipelines. Cortical thickness was measured as the distance from the gray matter–white matter boundary to the perpendicular pial surface. Mean regional cortical thickness values were then extracted for the Desikan-Killiany ROIs.

Regions-of-interest

A composite tau-PET region-of-interest (ROI) was created, often referred to as the temporal meta-ROI (amygdala, inferior/middle temporal gyri, fusiform gyrus, and parahippocampal gyrus)[30]. Because tau-PET uptake in the temporal meta-ROI has consistently been shown to

© 2024 Groot C et al. *JAMA Neurology.*

be the most accurate reflection of overall tau load[7,8,22,23,25,31-33], this measure was used as the primary measure of tau-PET in the main text while results using alternative regions are presented in eFigures-2 and 3. Aβ-PET levels were assessed within a global cortical mask and expressed in the Centiloids[34]. We computed cortical thickness in an "AD-signature" region (i.e., parahippocampal gyrus, inferior temporal, middle temporal, inferior parietal, fusiform, and precuneus)[35], which was selected as the primary metric for MRI as it has been shown to accurately cover areas known to experience cortical thinning in neurodegenerative diseases[35]. As for tau-PET, alternative ROIs for MRI are presented in eFigures-2 and 3.

Visual reads

Tau-PET positivity in the neocortex was assessed on color-scaled tau-PET SUVR images by an accredited rater (R.S. or C.G.). A positive read relied on tau-PET uptake in neocortical areas beyond the medial temporal lobes (i.e. spreading at least to the inferior/middle temporal gyrus), and these tau-PET visual reads were used in the main analyses. Results obtained using an alternative tau-PET positivity read (also allowing for positivity in the medial temporal lobes alone) are presented in eFigures-2 and 3. Aβ-PET SUVR images were visually assessed for global amyloid positivity by an accredited rater (L.E.C., V.G. or C.G.) based on established criteria for visual reads for [^{11}C]PiB[33], [^{18}F]florbetapir[34] and [^{18}F]flutemetamol[35]. MRI scans were visually assessed by an accredited rater (S.E.M., G.F. or C.G.) using the medial temporal lobe atrophy (MTA) rating scale and scores were converted into dichotomized MTA positivity (normal/abnormal) based on age-normalized criteria[36,37]. Results obtained using an alternative measure (global cortical atrophy; GCA) are presented in eFigures-2 and 3.

For each modality, we randomly selected 30 cases, which were then scored by two assessors. Cohen's kappa was then used to determine the inter-rater agreement, which ranged from moderate (0.59[0.32-0.86] for GCA score on MRI) to near perfect (0.93[0.79-1.00] for neocortical tau-PET; eTable 2).

© 2024 Groot C et al. *JAMA Neurology.*

Modality	Region	Dementia	Youden index cutpoint[a]	Threshold 2SD>Mean[b]	Metric
**Tau-PET**	Entorhinal cortex + amygdala	All-cause	1.37	1.30	SUVR
		AD	1.34		
	Temporal meta-region[1]	All-cause	1.41	1.35	SUVR
		AD	1.38		
	Braak V-VI[2]	All-cause	1.22	1.21	SUVR
		AD	1.22		
**Aβ-PET**	Global neocortex	All-cause	29.83	27	Centiloid
		AD	30.47		
**MRI**	Entorhinal cortex	All-cause	2.91	2.70	Cortical thickness, mm
		AD	2.93		
	AD-signature region[3]	All-cause	2.31	2.28	Cortical thickness, mm
		AD	2.36		
	Whole brain cortex	All-cause	2.24	2.17	Cortical thickness, mm
		AD	2.24		

eTable 1. Thresholds for positivity across modalities

SUVR–standardized uptake value ratio, cc–cubic centimeter, mm–millimeter. AD–Alzheimer's disease. 1-amygdala, inferior/middle temporal gyri, fusiform gyrus, and parahippocampal gyrus. 2-anterior cingulate, inferior frontal cortex, inferior parietal cortex, insular cortex, lateral occipital cortex, lingual gyrus, medial occipital cortex, middle frontal cortex, orbitofrontal cortex, paracentral cortex, precentral cortex, precuneus, postcentral cortex, posterior cingulate, superior frontal cortex, superior parietal cortex, superior temporal gyrus, and supramarginal gyrus. 3-parahippocampal gyrus, inferior temporal, middle temporal, inferior parietal, fusiform, and precuneus. a–threshold was determined based on the Youden index, i.e., the optimal cut-point to distinguish stable MCI from progressors to dementia, using the "cutpointr" packed in R, b–threshold was determined using a cognitively unimpaired control group comprised of BioFINDER-2 and ADNI participants as the reference and positivity on metrics in the main sample was set at mean+/-2SD within that control group (as previously described[7]).

© 2024 Groot C et al. *JAMA Neurology.*

Modality	Measure	Cohen's kappa	Cohen's kappa, 95%CI*	Agreement	N
**Tau-PET**	Medialtemporal tau	0.74	0.51-0.97	Substantial	30
**Tau-PET**	Neocortical tau	0.93	0.79-1.00*	Near perfect	30
**Aβ-PET**	Global neocortical amyloid	0.80	0.59-1.00*	Substantial	30
**MRI**	Medialtemporal atrophy	0.68	0.40-0.97	Substantial	30
**MRI**	Global cortical atrophy	0.59	0.32-0.86	Moderate	30

***95%CI deprecated at 1**

eTable 2. Inter-rater agreement for tau-PET, amyloid-PET and MRI visual reads

Two ratings were performed on 30 randomly sampled cases. Raters for Tau-PET: R.S., C.G. Raters for Aβ-PET: L.E.C., V.G., C.G. Raters for MRI: S.E.M.,G.B.F.,C.G.

© 2024 Groot C et al. *JAMA Neurology.*

Discovery cohort

	All-cause dementia						AD dementia					
	Accuracy	Specificity	Sensitivity	AUC	PPV	NPV	Accuracy	Specificity	Sensitivity	AUC	PPV	NPV
**Demographics**	0.70[0.63-0.76]	0.75[0.60-0.85]	0.62[0.48-0.76]	0.71[0.65-0.77]	0.56[0.47-0.65]	0.79[0.73-0.84]	0.76[0.65-0.81]	0.79[0.62-0.88]	0.66[0.51-0.83]	0.75[0.69-0.82]	0.50[0.40-0.60]	0.87[0.83-0.92]
**Tau-PET**	0.70[0.64-0.76]	0.66[0.56-0.79]	0.77[0.61-0.86]	0.75[0.70-0.80]	0.52[0.44-0.59]	0.85[0.80-0.91]	0.78[0.70-0.84]	0.76[0.64-0.84]	0.85[0.73-0.94]	0.84[0.79-0.89]	0.54[0.44-0.63]	0.92[0.88-0.96]
**Aβ-PET**	0.66[0.60-0.76]	0.57[0.48-0.89]	0.82[0.47-0.90]	0.73[0.68-0.79]	0.48[0.41-0.55]	0.86[0.80-0.92]	0.71[0.65-0.84]	0.63[0.55-0.88]	0.94[0.68-0.99]	0.83[0.78-0.88]	0.44[0.36-0.52]	0.97[0.94-1.00]
**MRI**	0.71[0.65-0.76]	0.71[0.58-0.85]	0.72[0.55-0.83]	0.74[0.68-0.80]	0.55[0.47-0.63]	0.83[0.77-0.88]	0.77[0.63-0.83]	0.82[0.56-0.89]	0.66[0.52-0.89]	0.76[0.70-0.83]	0.55[0.43-0.66]	0.87[0.82-0.91]
**Tau-PET visual read**	0.69[0.63-0.75]	0.67[0.56-0.76]	0.75[0.65-0.85]	0.74[0.68-0.79]	0.53[0.45-0.61]	0.83[0.78-0.89]	0.75[0.68-0.81]	0.71[0.61-0.80]	0.87[0.77-0.96]	0.83[0.78-0.88]	0.48[0.39-0.57]	0.94[0.90-0.98]
**Amyloid β-PET visual read**	0.64[0.58-0.77]	0.55[0.46-0.90]	0.80[0.43-0.90]	0.72[0.66-0.78]	0.45[0.39-0.52]	0.86[0.80-0.92]	0.72[0.60-0.84]	0.69[0.49-0.90]	0.83[0.59-0.97]	0.81[0.76-0.87]	0.41[0.34-0.49]	0.94[0.90-0.98]
**MRI visual read**	0.69[0.62-0.74]	0.67[0.53-0.76]	0.74[0.62-0.85]	0.73[0.68-0.79]	0.51[0.44-0.59]	0.83[0.78-0.89]	0.70[0.60-0.81]	0.68[0.51-0.89]	0.77[0.51-0.92]	0.77[0.71-0.83]	0.41[0.33-0.50]	0.90[0.85-0.95]

© 2024 Groot C et al. *JAMA Neurology.*

Validation cohort

	All-cause dementia						AD dementia					
	Accuracy	Specificity	Sensitivity	AUC	PPV	NPV	Accuracy	Specificity	Sensitivity	AUC	PPV	NPV
**Demographics**	0.77[0.68-0.88]	0.77[0.66-0.92]	0.79[0.53-0.95]	0.73[0.59-0.88]	0.36[0.21-0.50]	0.95[0.90-1.00]	0.71[0.51-0.77]	0.75[0.40-0.87]	0.58[0.38-0.85]	0.66[0.59-0.74]	0.40[0.31-0.50]	0.84[0.79-0.89]
**Tau-PET**	0.80[0.69-0.90]	0.78[0.64-0.91]	0.95[0.74-1.00]	0.85[0.75-0.95]	0.38[0.24-0.52]	0.99[0.96-1.01]	0.73[0.63-0.79]	0.71[0.55-0.80]	0.82[0.69-0.93]	0.79[0.73-0.84]	0.46[0.37-0.55]	0.91[0.87-0.96]
**Aβ-PET**	0.76[0.55-0.89]	0.74[0.47-0.92]	0.84[0.58-1.00]	0.79[0.68-0.90]	0.33[0.20-0.47]	0.94[0.89-1.00]	0.64[0.58-0.76]	0.54[0.46-0.76]	0.94[0.72-0.99]	0.78[0.72-0.83]	0.39[0.31-0.46]	0.97[0.93-1.00]
**MRI**	0.74[0.64-0.87]	0.71[0.58-0.87]	0.95[0.74-1.00]	0.83[0.74-0.92]	0.35[0.22-0.48]	0.99[0.96-1.02]	0.69[0.54-0.80]	0.70[0.44-0.94]	0.65[0.34-0.89]	0.69[0.62-0.76]	0.49[0.37-0.62]	0.83[0.78-0.88]
**Tau-PET visual read**	0.81[0.67-0.91]	0.80[0.60-0.94]	0.89[0.68-1.00]	0.85[0.75-0.94]	0.41[0.26-0.57]	0.96[0.92-1.00]	0.68[0.60-0.75]	0.62[0.50-0.73]	0.86[0.75-0.94]	0.76[0.71-0.82]	0.41[0.33-0.49]	0.93[0.89-0.97]
**Amyloid β-PET visual read**	0.72[0.61-0.91]	0.67[0.54-0.94]	0.89[0.63-1.00]	0.81[0.70-0.92]	0.32[0.20-0.44]	0.98[0.95-1.02]	0.69[0.56-0.79]	0.67[0.45-0.87]	0.79[0.54-0.96]	0.76[0.70-0.82]	0.37[0.29-0.44]	0.93[0.89-0.98]
**MRI visual read**	0.74[0.63-0.85]	0.73[0.59-0.91]	0.79[0.53-0.95]	0.74[0.61-0.87]	0.34[0.20-0.48]	0.94[0.89-1.00]	0.63[0.53-0.76]	0.59[0.42-0.82]	0.76[0.46-0.90]	0.69[0.62-0.76]	0.36[0.28-0.43]	0.88[0.83-0.93]

eTable 3. Receiver operating curve analysis assessing the predictive power of neuroimaging to detect progressors among individuals with MCI

All values displayed are statistic(95% confidence interval). The models for neuroimaging markers also included the demographics and every model were corrected for follow-up time and cohort effects. Between brackets are the 95% confidence intervals. AD – Alzheimer’s disease, AUC-area under the curve, PPV-positive predictive value, NPV-negative predictive value.

© 2024 Groot C et al. *JAMA Neurology.*

	Validation cohort							
	Total sample	Stable MCI	Progressors all-cause dementia	p vs stable MCI	Progressors AD dementia	p vs stable MCI	Progressors Dementia-other	p vs stable MCI
**n**	117	98	19		16		3	
**Age**	72.7 (7.1)	72.2 (7.0)	75.1 (7.2)	0.12	75.8 (7.1)	0.06	71.0 (8.2)	0.76
**Sex, male**	67 (57.3)	56 (57.1)	11 (57.9)	1	9 (56.2)	1	2 (66.7)	1
**Education. years**	16.4 (2.7)	16.2 (2.8)	17.3 (2.1)	0.11	17.3 (2.1)	0.14	17.3 (2.3)	0.49
***APOEε*4**	37 (36.3)	32 (36.8)	5 (33.3)	1	5 (41.7)	0.99	0 (0.0)	0.49
**MMSE**	27.8 (1.9)	28.0 (1.8)	27.1 (2.1)	0.05	27.4 (2.0)	0.28	25.0 (2.0)	0.01
**Follow-up time, years**	2.2 (1.2)	2.2 (1.3)	2.2 (1.0)	0.91	2.2 (1.0)	0.98	2.0 (1.0)	0.81
**Temporal meta-region tau, SUVR**	1.3 (0.3)	1.3 (0.2)	1.5 (0.3)	**0.00**	1.5 (0.3)	**0.00**	1.6 (0.4)	0.03
**Tau-PET visual read positive**	31 (26.5)	18 (18.4)	13 (68.4)	**0.00**	11 (68.8)	**0.00**	2 (66.7)	0.18
**Aβ-PET, Centiloids**	46.8 (55.1)	39.8 (52.2)	82.8 (56.8)	**0.00**	82.0 (56.0)	**0.00**	87.4 (74.0)	0.13
**Aβ-PET visual read positive**	55 (47.0)	39 (39.8)	16 (84.2)	**0.00**	14 (87.5)	**0.00**	2 (66.7)	0.74
**AD-signature cortical thickness, mm**	2.4 (0.1)	2.4 (0.1)	2.3 (0.1)	**0.00**	2.3 (0.1)	**0.00**	2.2 (0.1)	0.01
**Medial temporal atrophy visual read positive**	15 (12.9)	11 (11.3)	4 (21.1)	0.44	3 (18.8)	0.67	1 (33.3)	0.8

eTable 5. Baseline demographic and clinical characteristics of the validation cohort

Values are mean (SD) for continuous variables and N (%) for categorical variables. Differences in continuous variables between progressors and stable MCI were assessed using pairwise independent t-tests and by Fisher's exact tests for categorical variables. False-discovery rate corrected significant differences at α=0.05 between groups are displayed in **bold.** SUVR–standardized uptake value ratio, MMSE–mini mental state examination. The "progressors AD dementia" and "progressors Dementia-other" are subgroups of the "progressors all-cause dementia" group. Dementia-other: FTD n=1, VaD n=1, unknown etiology n=1. a–Values in these rows indicate N(% of group [column] from that cohort).

© 2024 Groot C et al. *JAMA Neurology.*

Discovery cohort

Base model

All-cause dementia

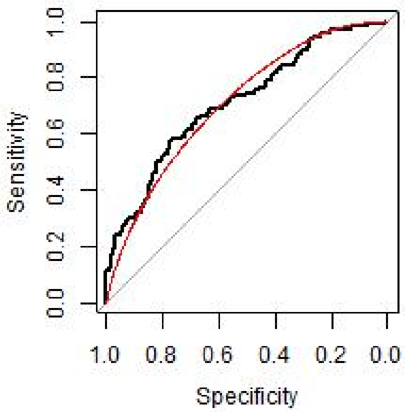

AD dementia

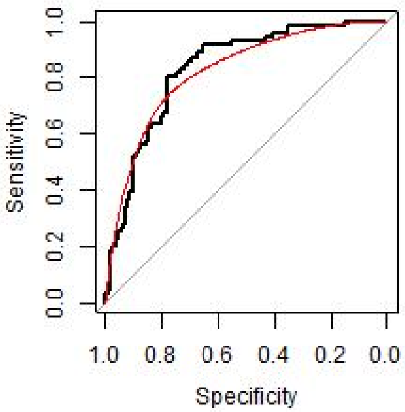

Temporal meta-ROI tau

All-cause dementia

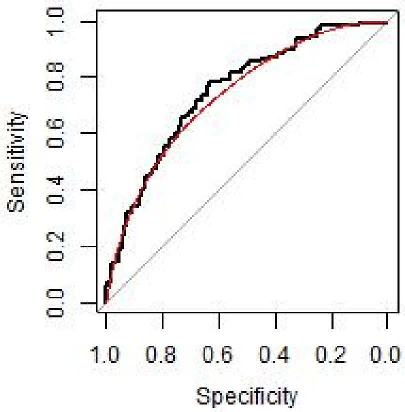

AD dementia

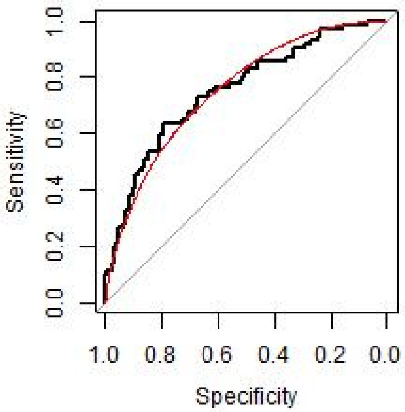

Tau-PET visual read

All-cause dementia

AD dementia

© 2024 Groot C et al. *JAMA Neurology.*

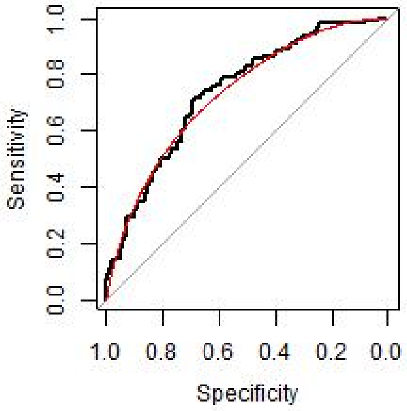

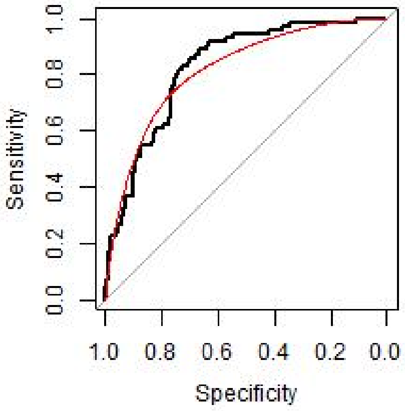

Centiloids

All-cause dementia

AD dementia

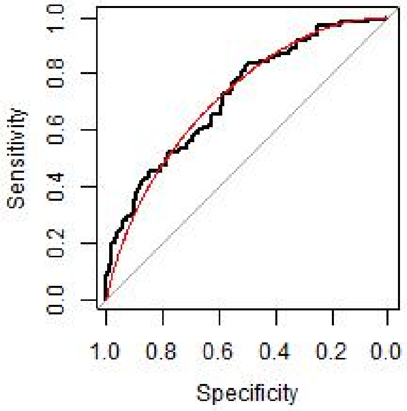

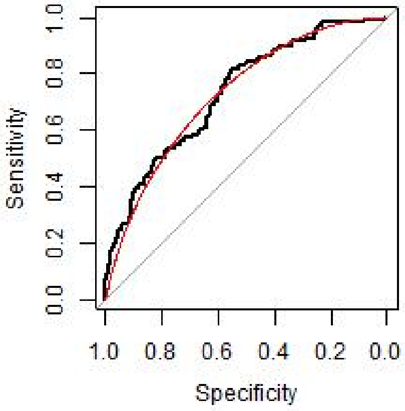

Aβ-PET visual read

All-cause dementia

AD dementia

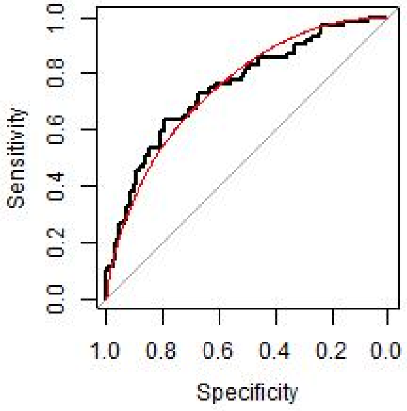

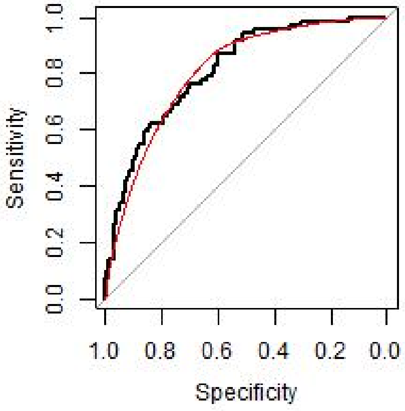

AD-signature thickness

© 2024 Groot C et al. *JAMA Neurology*.

All-cause dementia

AD dementia

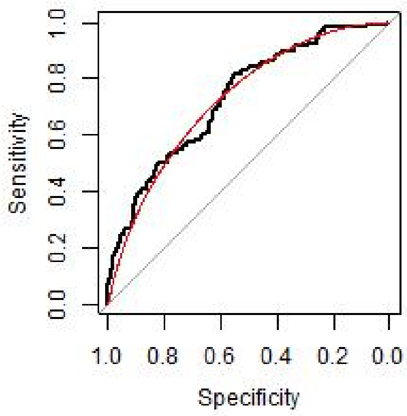

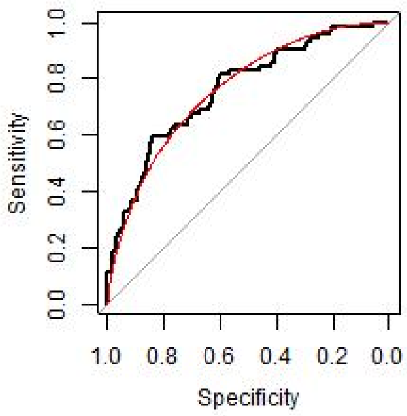

MTA visual read

All-cause dementia

AD dementia

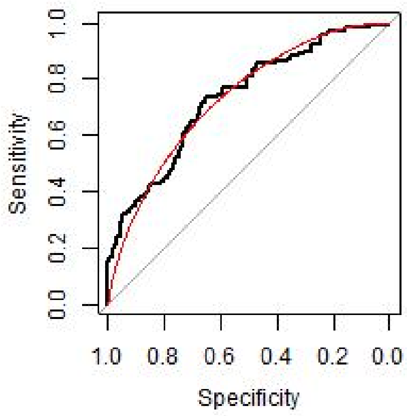

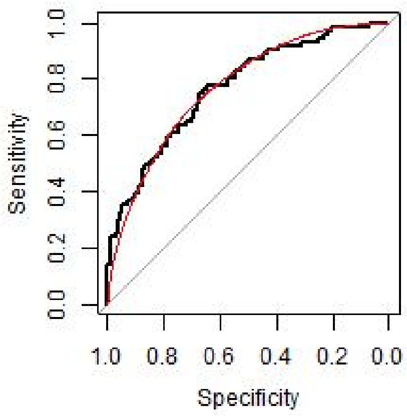

Validation cohort

Base model

All-cause dementia

AD dementia

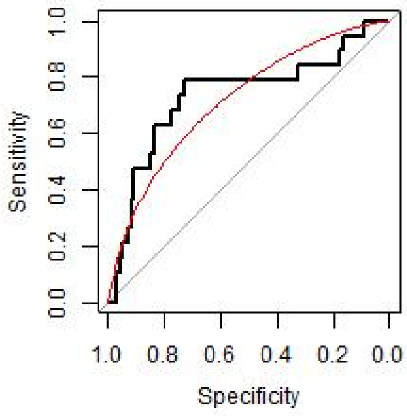

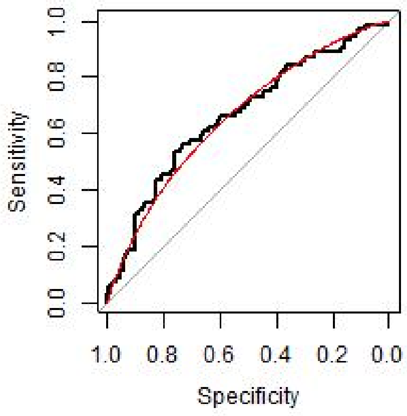

© 2024 Groot C et al. *JAMA Neurology*.

Temporal meta-ROI tau

All-cause dementia

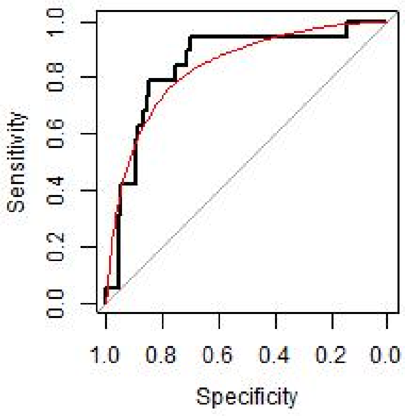

AD dementia

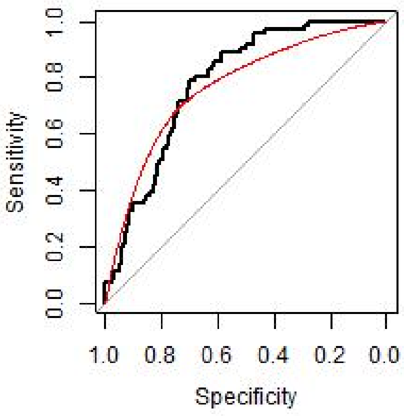

Tau-PET visual read

All-cause dementia

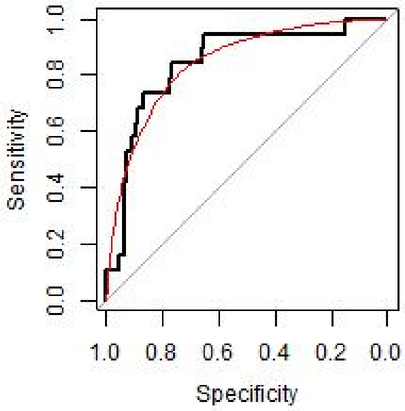

AD dementia

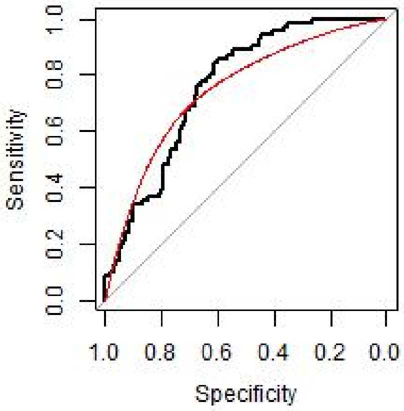

Centiloids

All-cause dementia

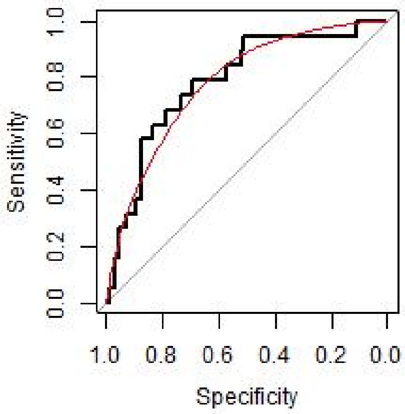

AD dementia

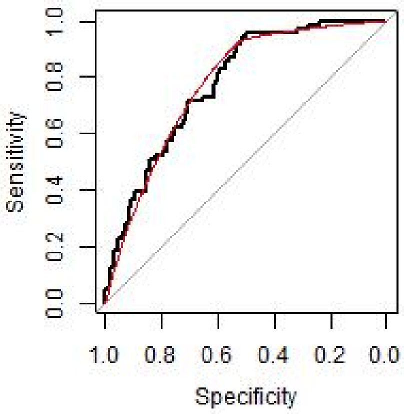

© 2024 Groot C et al. *JAMA Neurology*.

Aβ-PET visual read

All-cause dementia

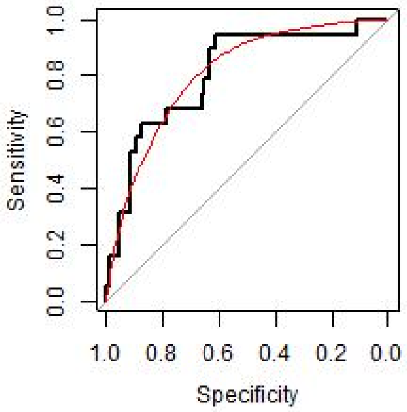

AD dementia

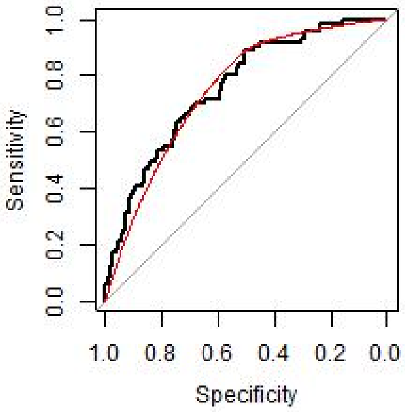

AD-signature thickness

All-cause dementia

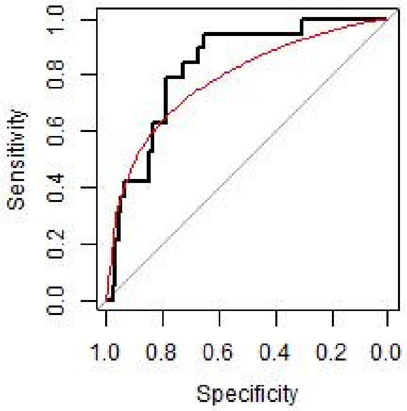

AD dementia

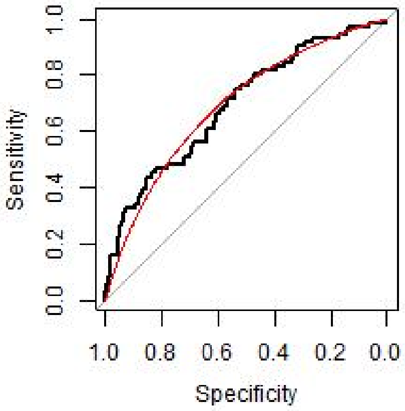

MTA visual read

All-cause dementia

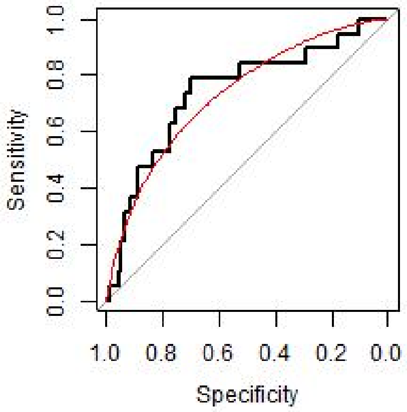

AD dementia

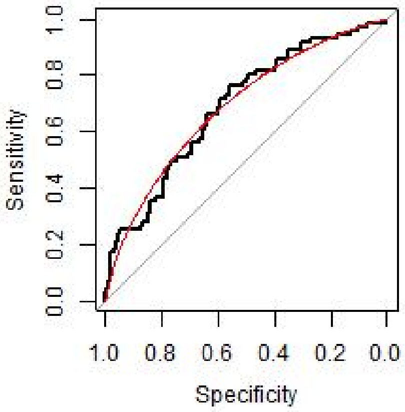

© 2024 Groot C et al. *JAMA Neurology.*

eFigure 1. Calibration plots for receiver operating curve analyses

The plots display the correspondence between the empirical receiver-operating characteristic (ROC; black) and model-based ROC (mROC; red). The overlap between these two lines indicates good calibration of the models. Model-based curves were generated using the mROC command from the “predtools” package for *R*.

© 2024 Groot C et al. *JAMA Neurology*.

Discovery cohort

Tau-PET

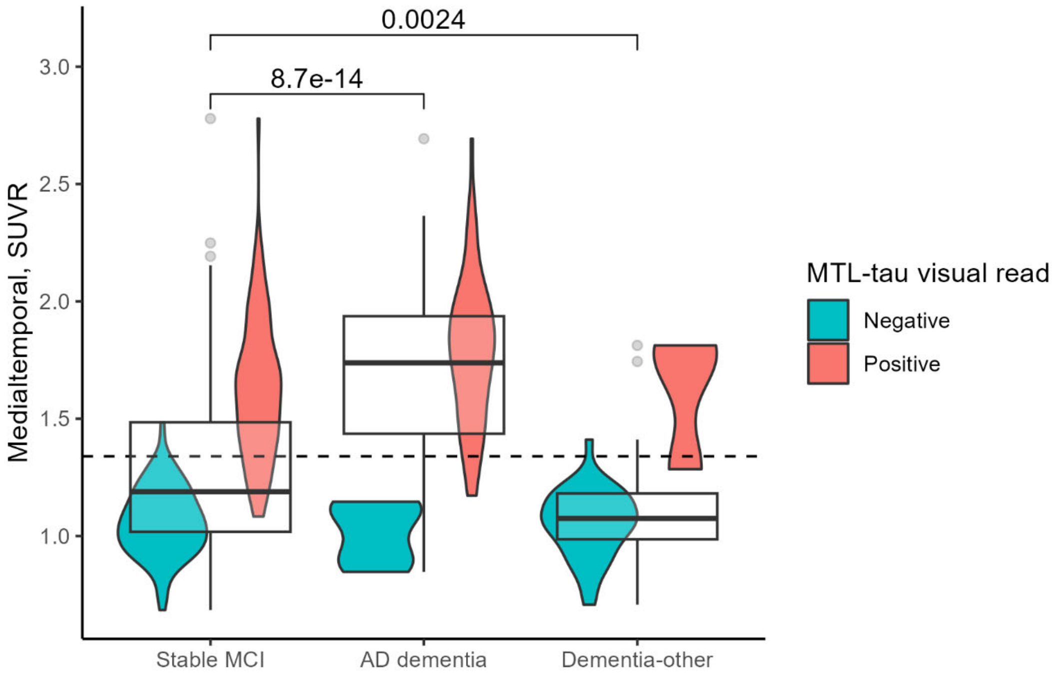

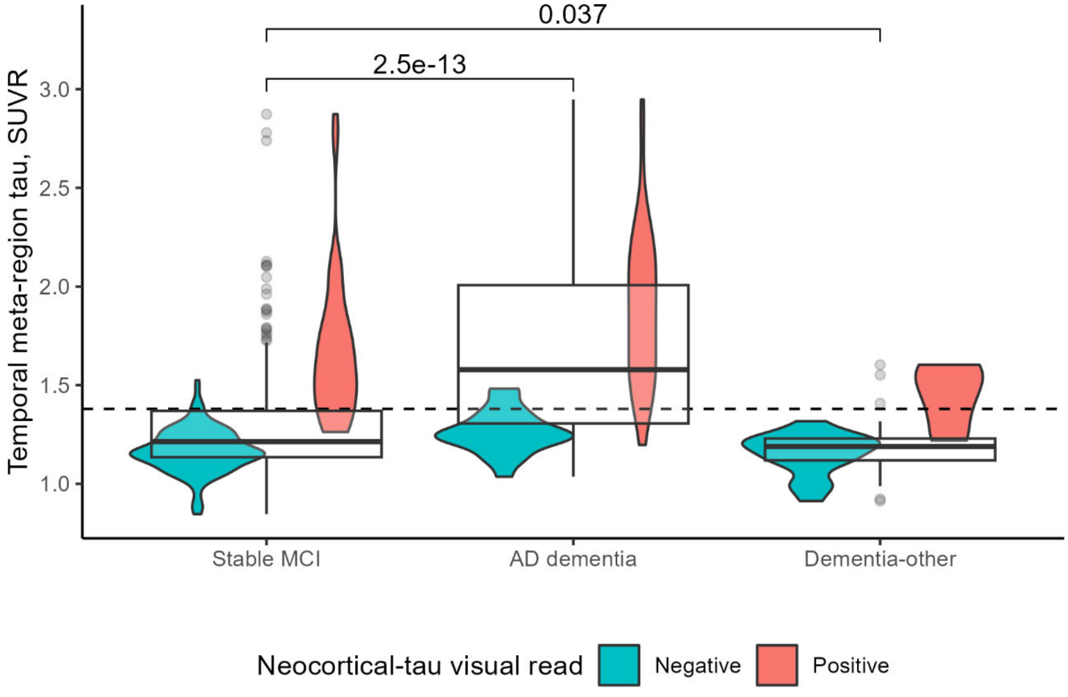

© 2024 Groot C et al. *JAMA Neurology.*

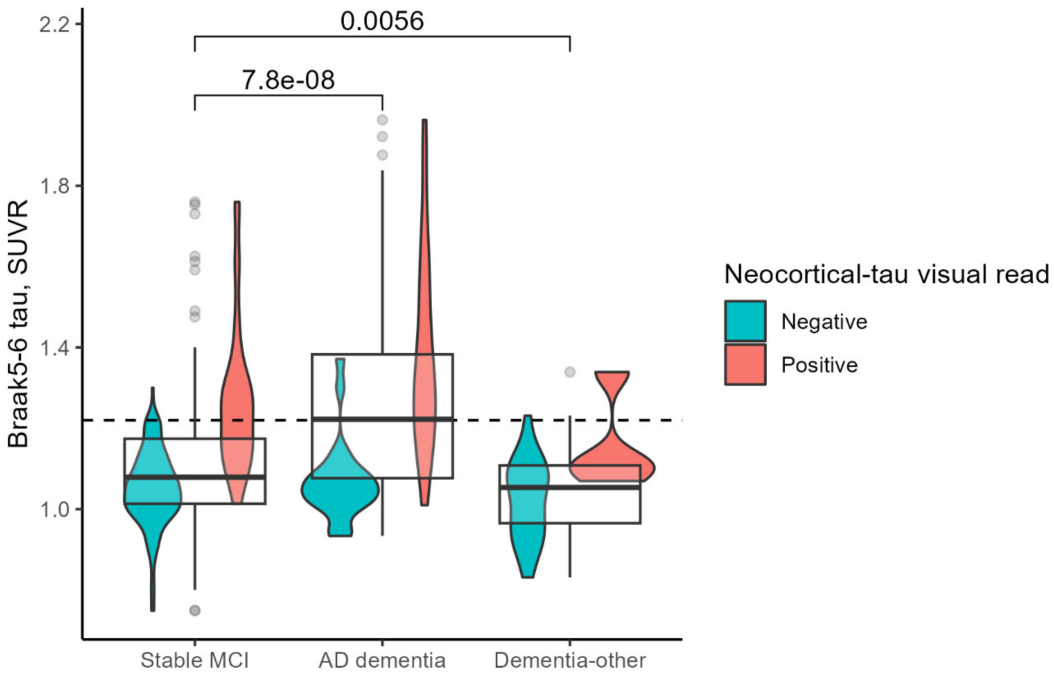

Amyloid-PET

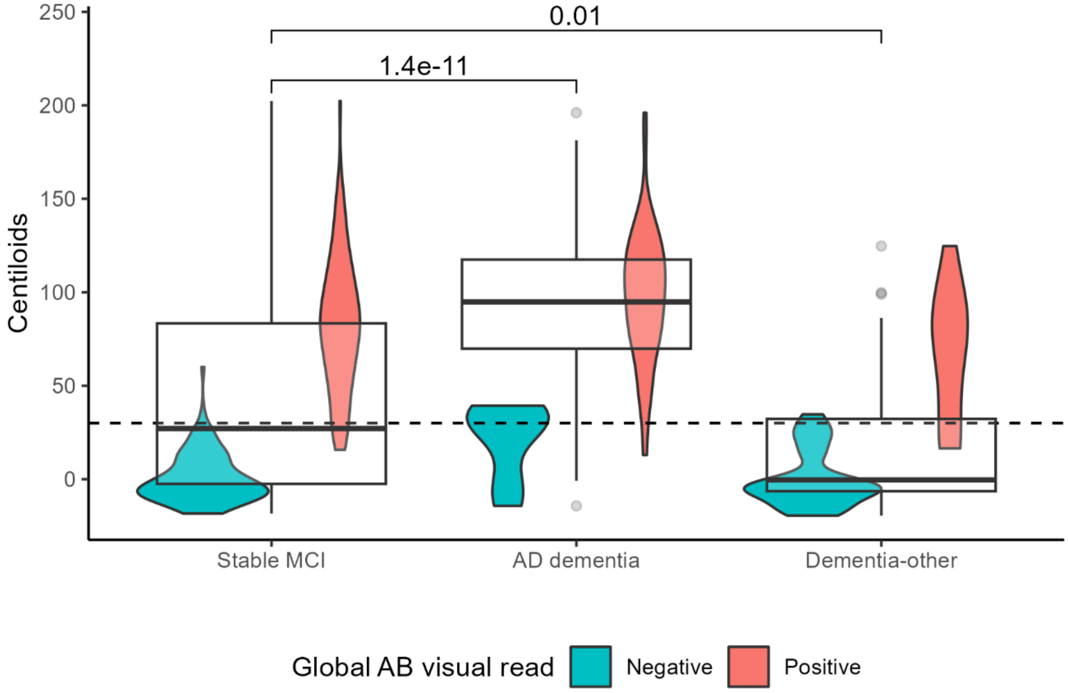

MRI

© 2024 Groot C et al. *JAMA Neurology*.

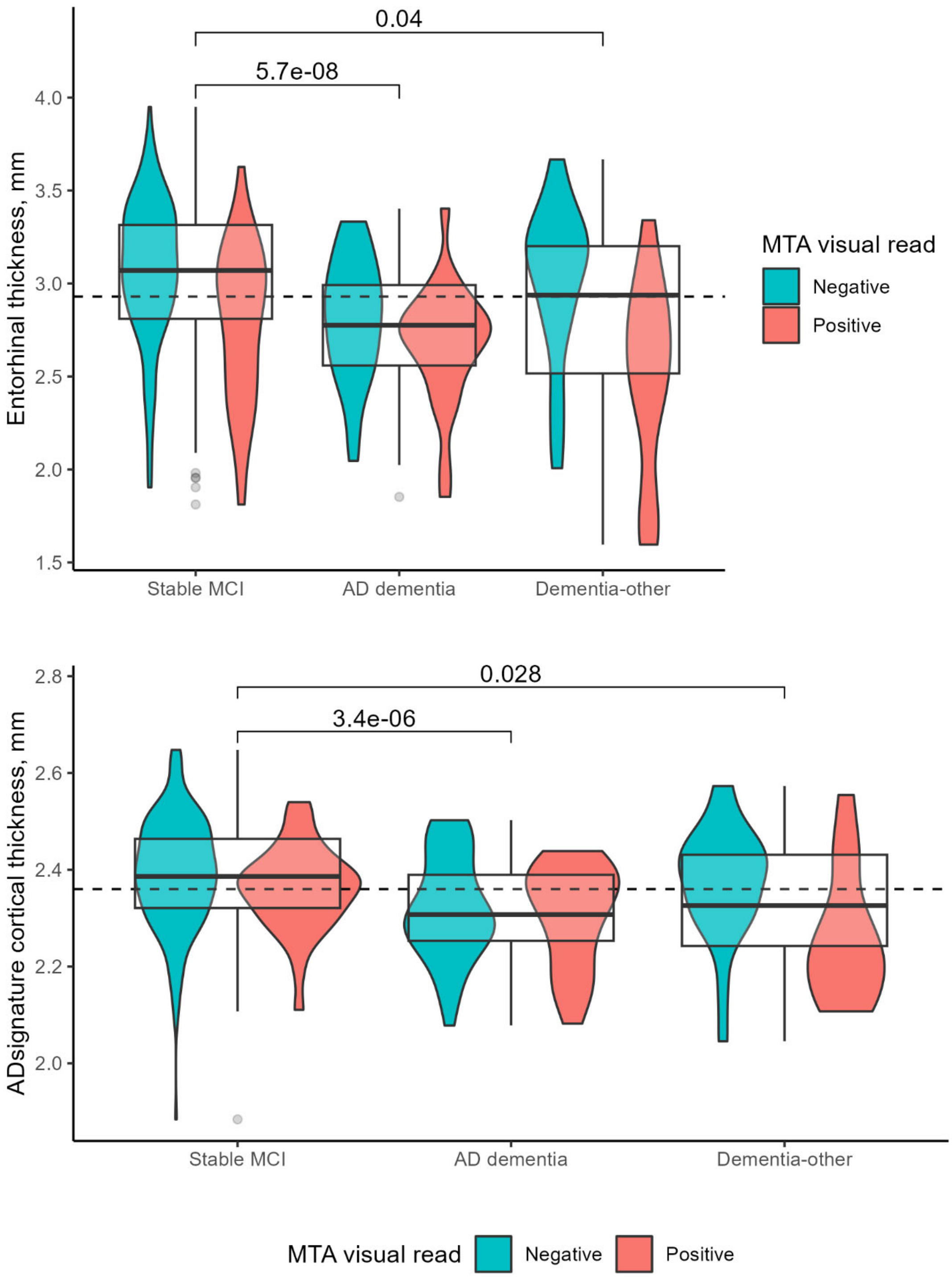

© 2024 Groot C et al. *JAMA Neurology*.

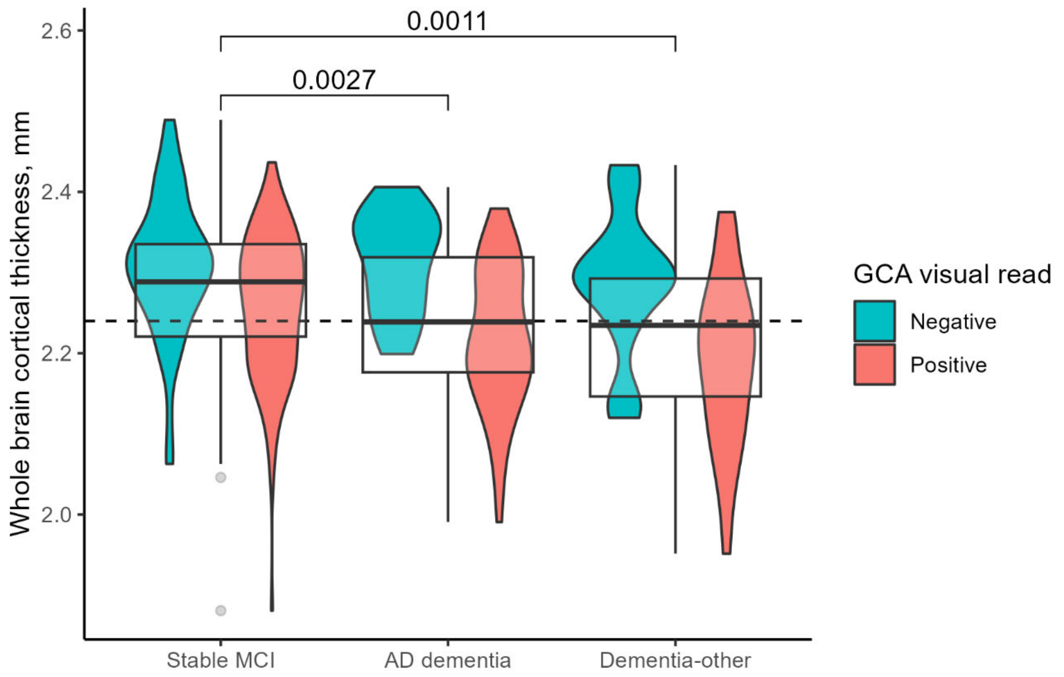

Validation cohort

Tau-PET

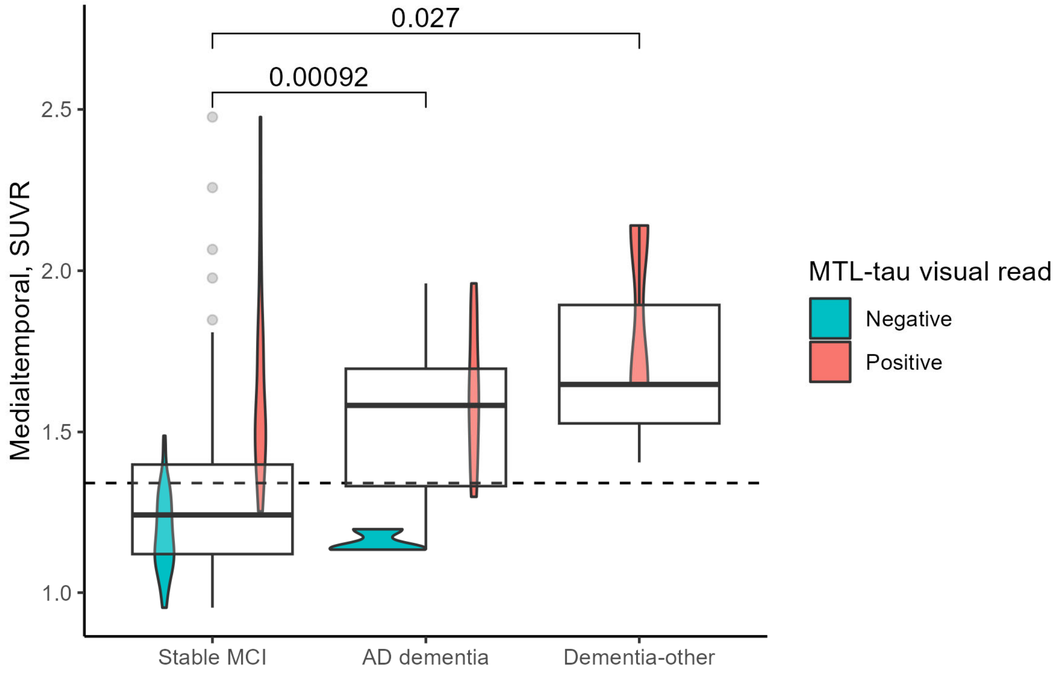

© 2024 Groot C et al. *JAMA Neurology*.

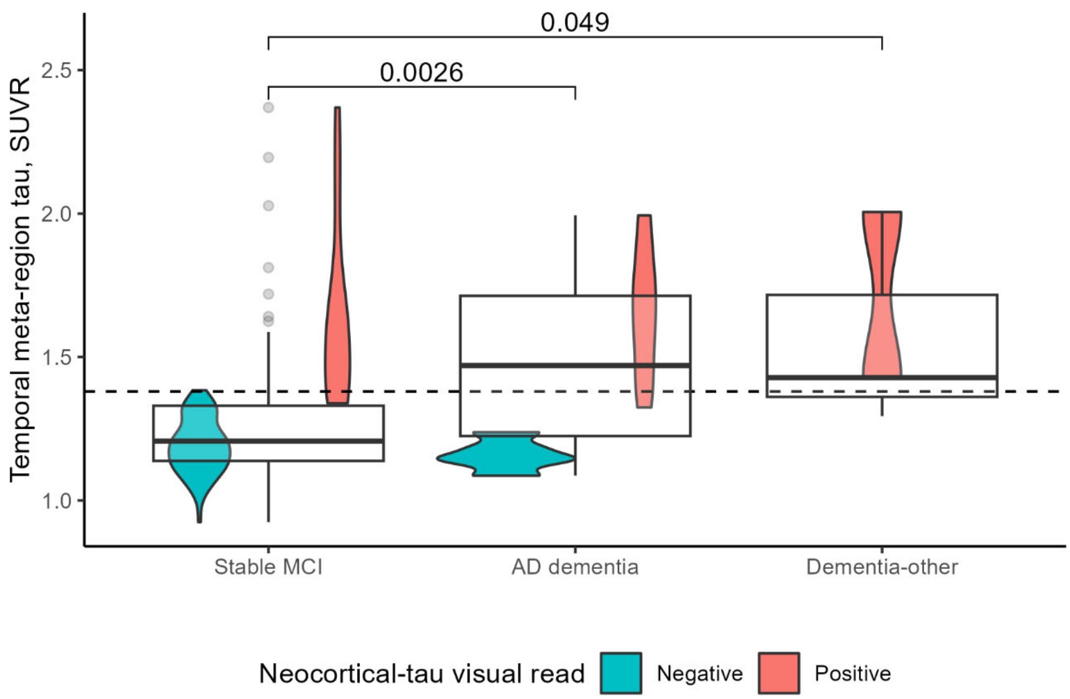

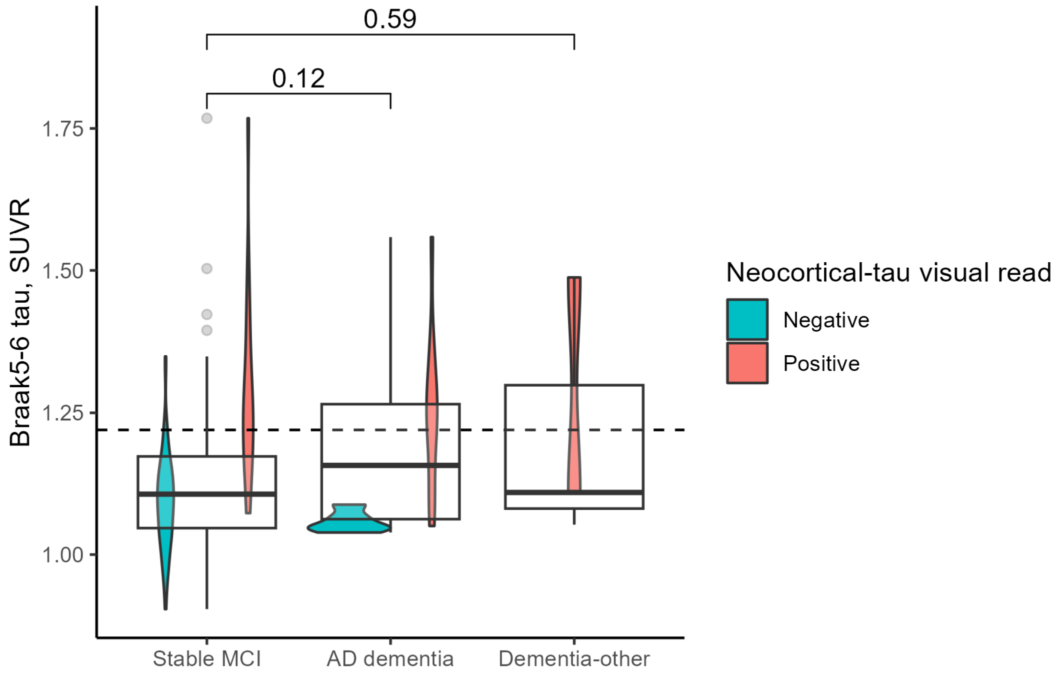

Amyloid-PET

© 2024 Groot C et al. *JAMA Neurology*.

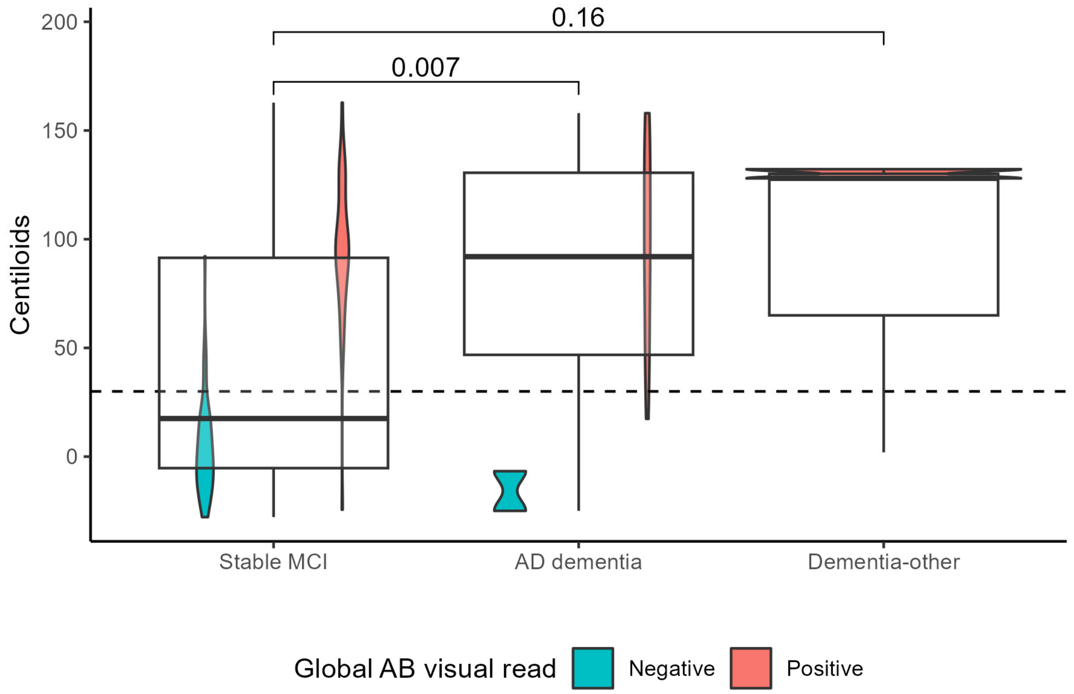

MRI

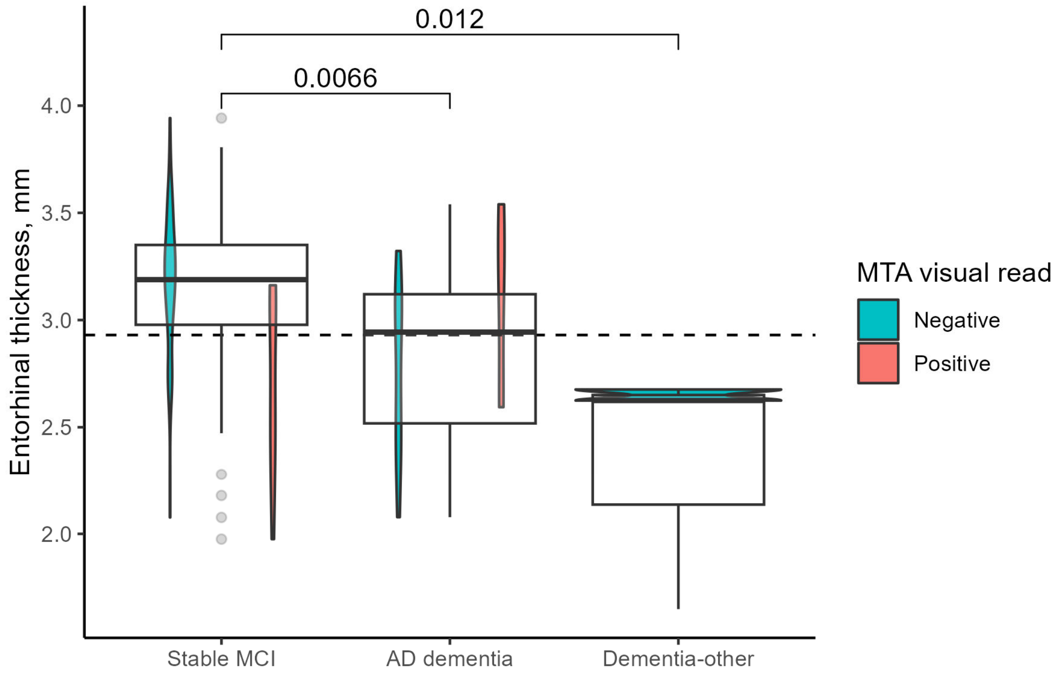

© 2024 Groot C et al. *JAMA Neurology.*

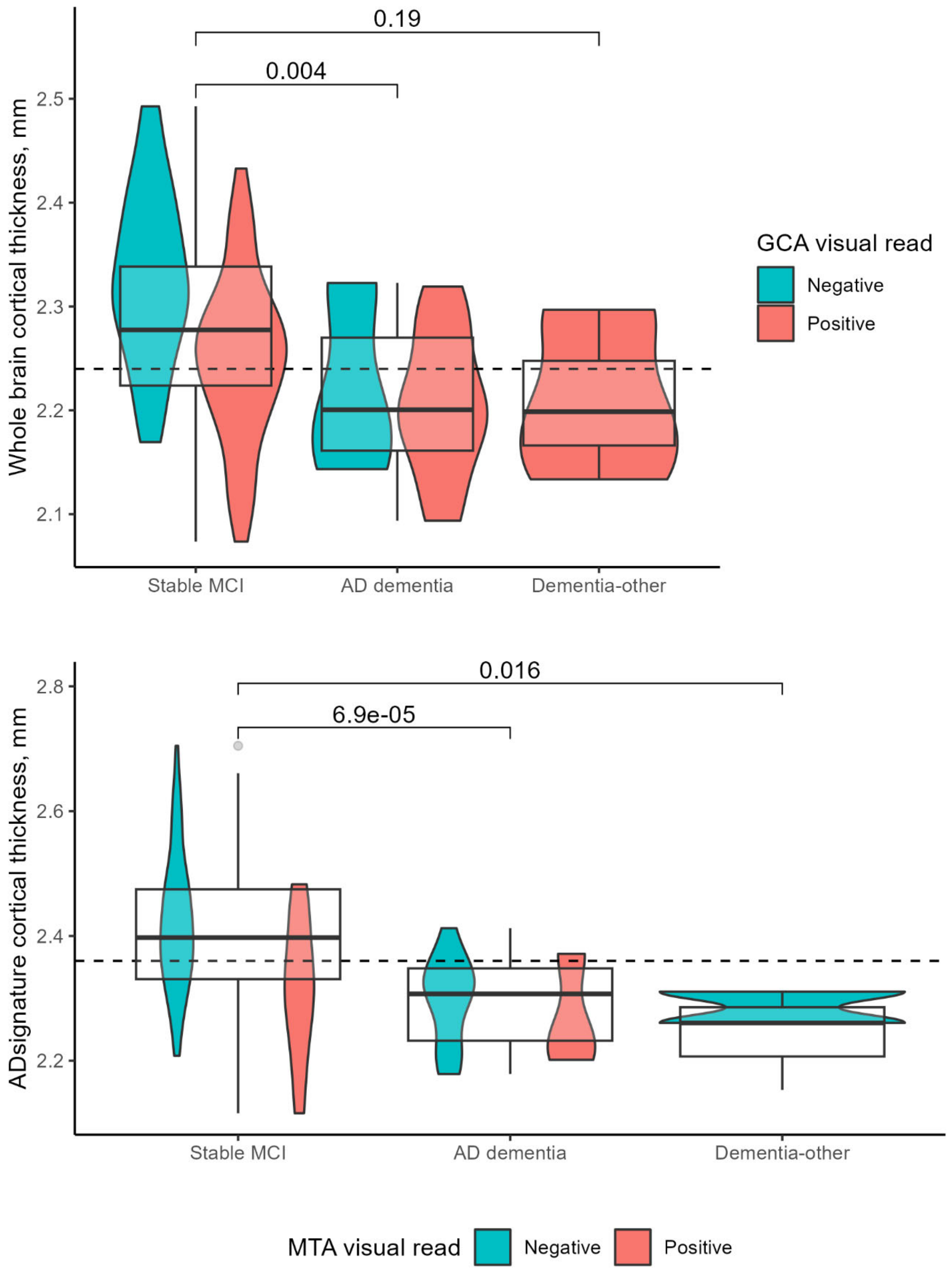

© 2024 Groot C et al. *JAMA Neurology*.

eFigure 2. Differences in tau-PET, Aβ-PET and MRI measures between individuals with stable MCI and progressors across alternative ROIs

As the predictive power to detect conversion of neuroimaging markers is inherently linked to which ROI is assessed, we explored alternative ROIs for tau-PET and MRI measures. Differences in tau-PET and MRI measures within the other ROIs between stable MCI and progressors are displayed in the boxplots and differences between groups were determined by independent sample t-tests and statistics displayed are p-values. Please note that the coloring of datapoints is dependent on various ROIs for visual reads, whichever visual read ROI most closely matched the ROI for the quantified measure. AD–Alzheimer's disease, MTA–medial temporal atrophy, SUVR–standardized uptake value ratio, mm–millimeter.

© 2024 Groot C et al. *JAMA Neurology.*

Discovery cohort

All-cause dementia

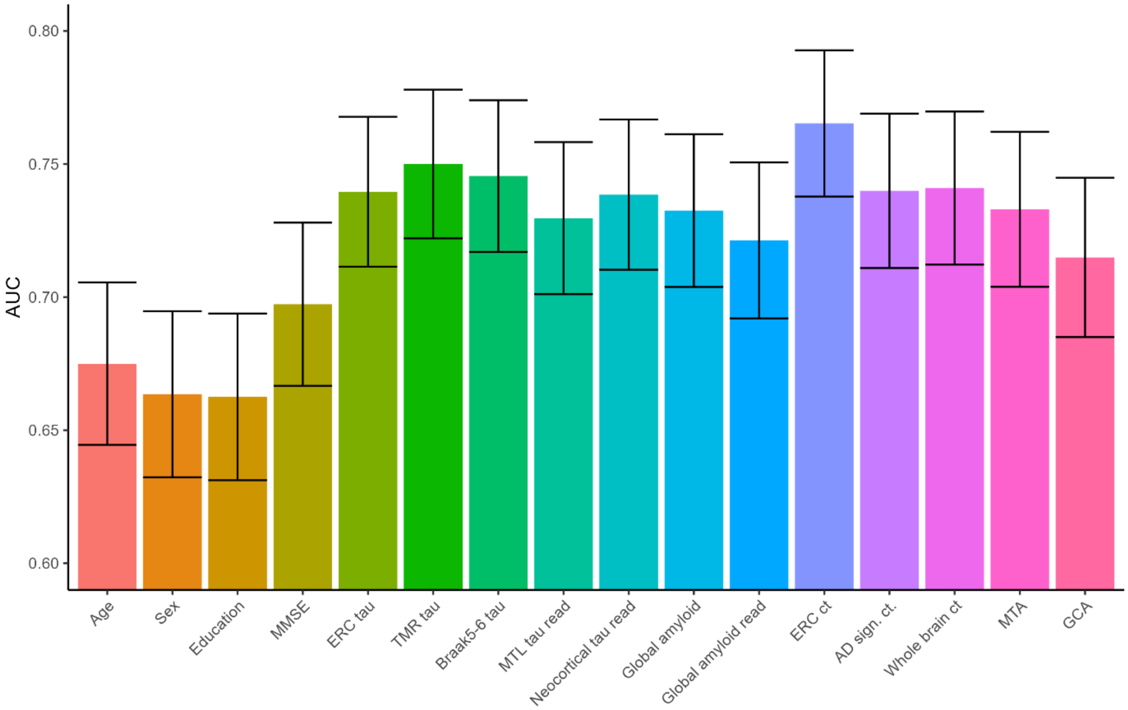

AD dementia

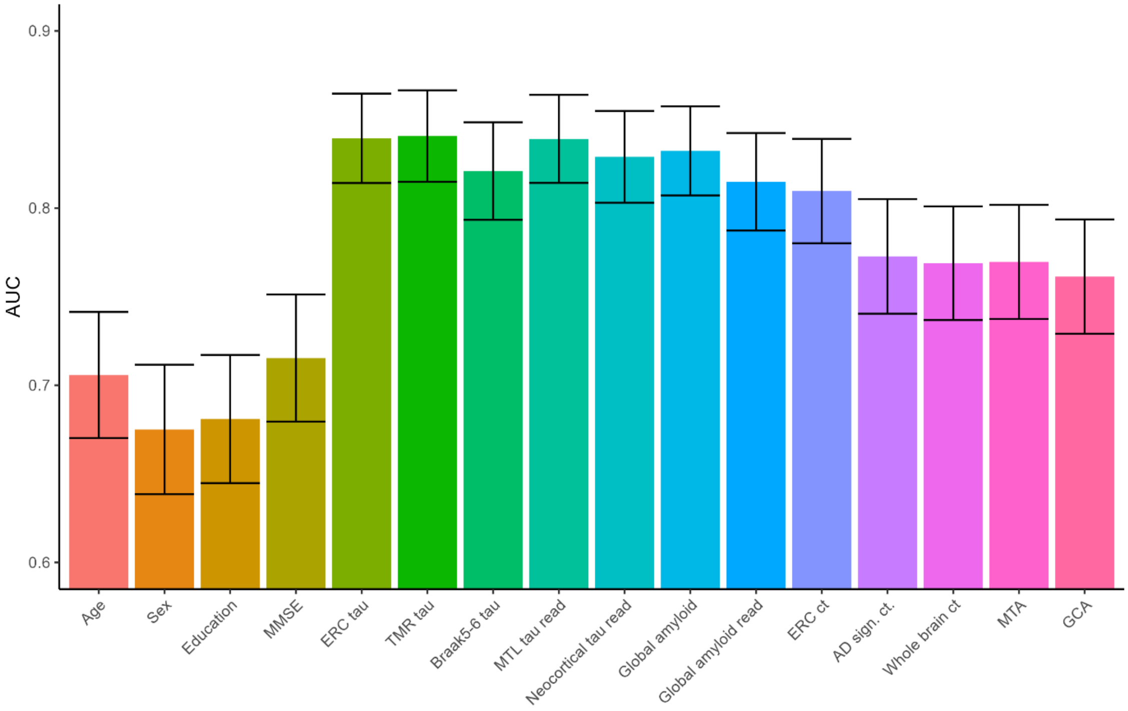

© 2024 Groot C et al. *JAMA Neurology*.

Validation cohort

All-cause dementia

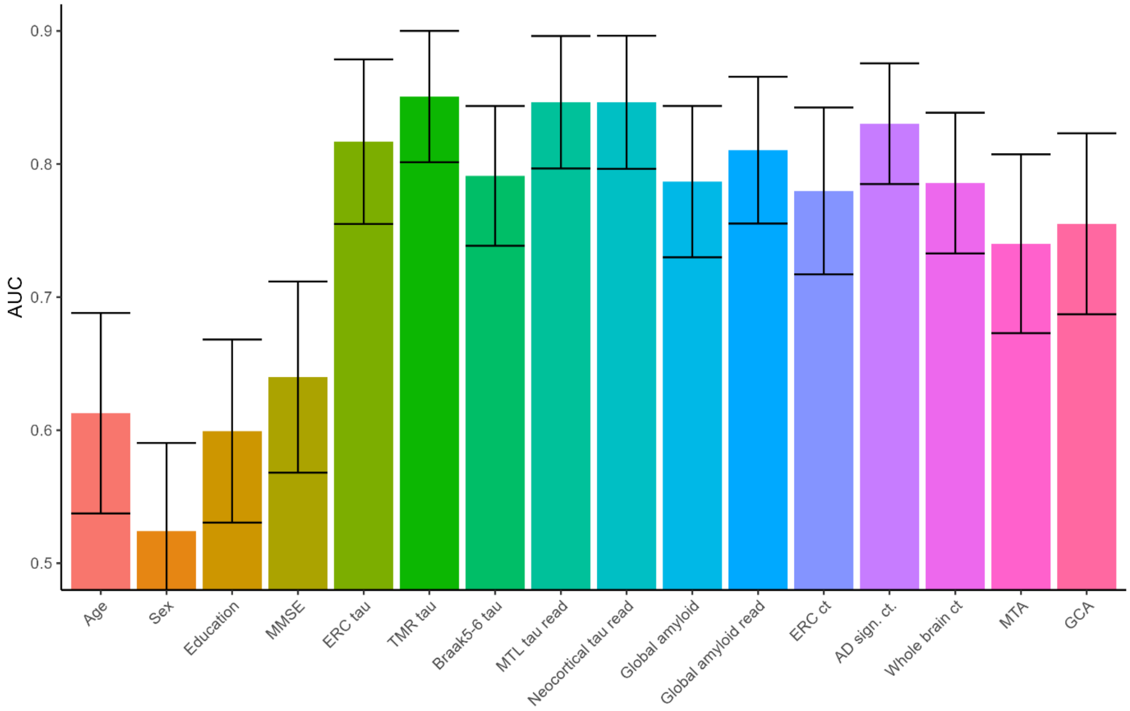

AD dementia

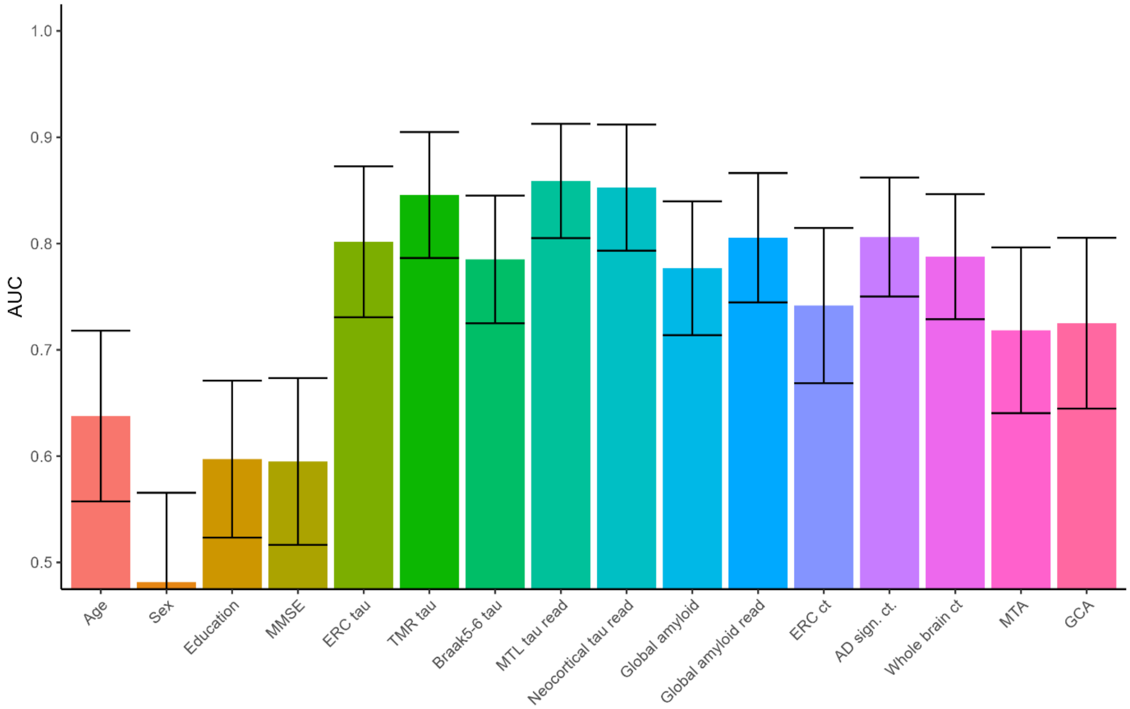

© 2024 Groot C et al. *JAMA Neurology*.

eFigure 3. Performance of all neuroimaging markers, including alternative ROIs, to detect progressors among individuals with MCI

The AUC to distinguish progressors from stable MCI associated within all ROIs are presented in the bar graph. AUC statistics were obtained from receiver operating characteristic analyses, adjusted for follow-up time and cohort. For the models assessing the neuroimaging markers, we additionally corrected for age, sex, education and MMSE. ERC tau–an unweighted average of the entorhinal cortex and amygdala, TMR tau–tau in a temporal meta-region (amygdala, inferior/middle temporal gyri, fusiform gyrus, and parahippocampal gyrus)[8], Braak 5/6 tau–composite ROI (anterior cingulate, inferior frontal cortex, inferior parietal cortex, insular cortex, lateral occipital cortex, lingual gyrus, medial occipital cortex, middle frontal cortex, orbitofrontal cortex, paracentral cortex, precentral cortex, precuneus, postcentral cortex, posterior cingulate, superior frontal cortex, superior parietal cortex, superior temporal gyrus, and supramarginal gyrus), MTL tau read–visual read for tau-PET within the medial temporal lobe, ERC ct–cortical thickness in the entorhinal cortex, AD sign. Ct.–cortical thickness in an "AD-signature" region (parahippocampal gyrus, inferior temporal, middle temporal, inferior parietal, fusiform, and precuneus)[9], whole brain ct–a whole brain composite region (comprising all neocortical regions), MTA–medial temporal lobe atrophy visual read (normal/abnormal), GCA–global cortical atrophy visual read (normal/abnormal).

© 2024 Groot C et al. *JAMA Neurology.*

Discovery cohort

All-cause dementia

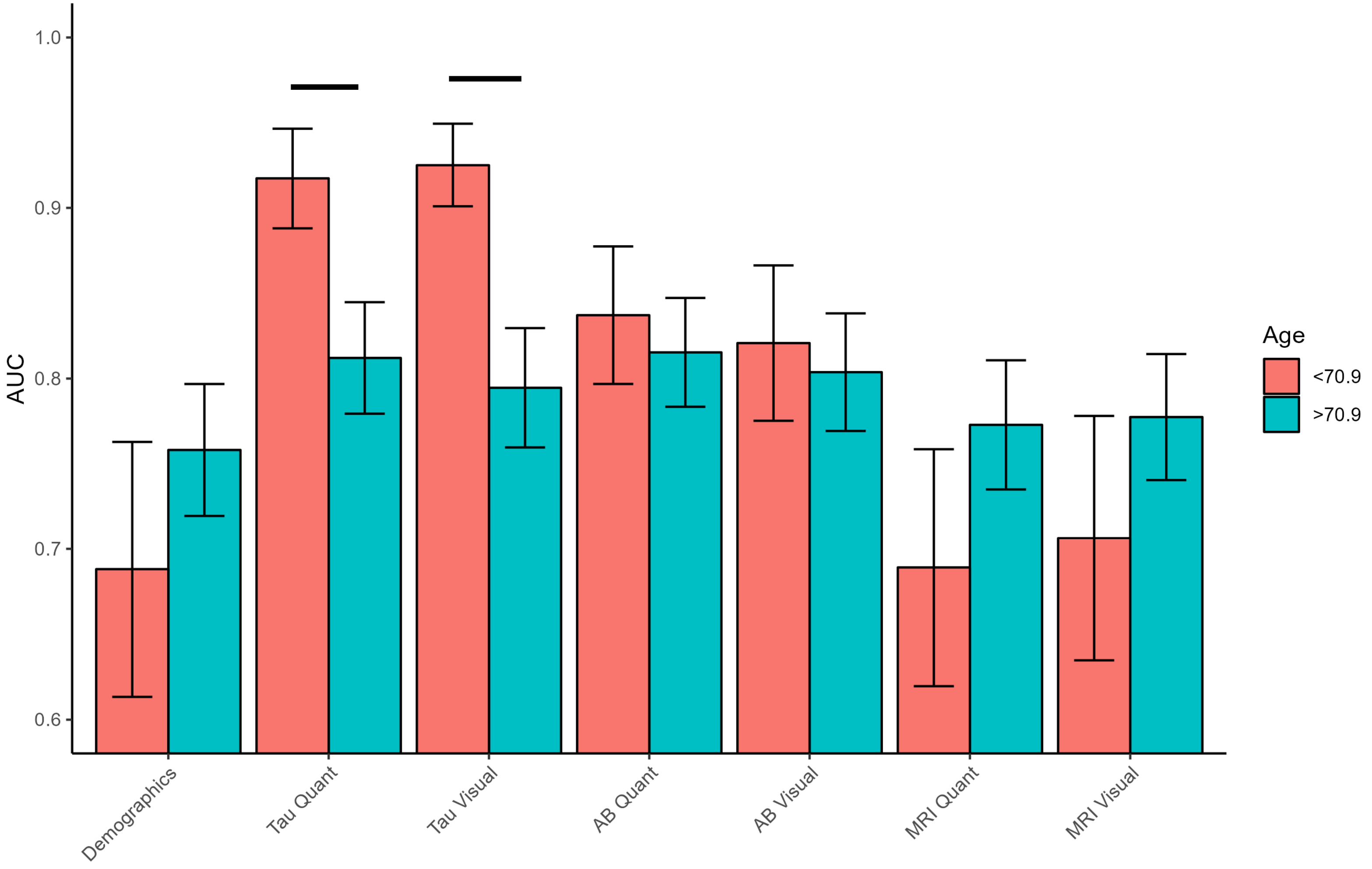

© 2024 Groot C et al. *JAMA Neurology.*

AD dementia

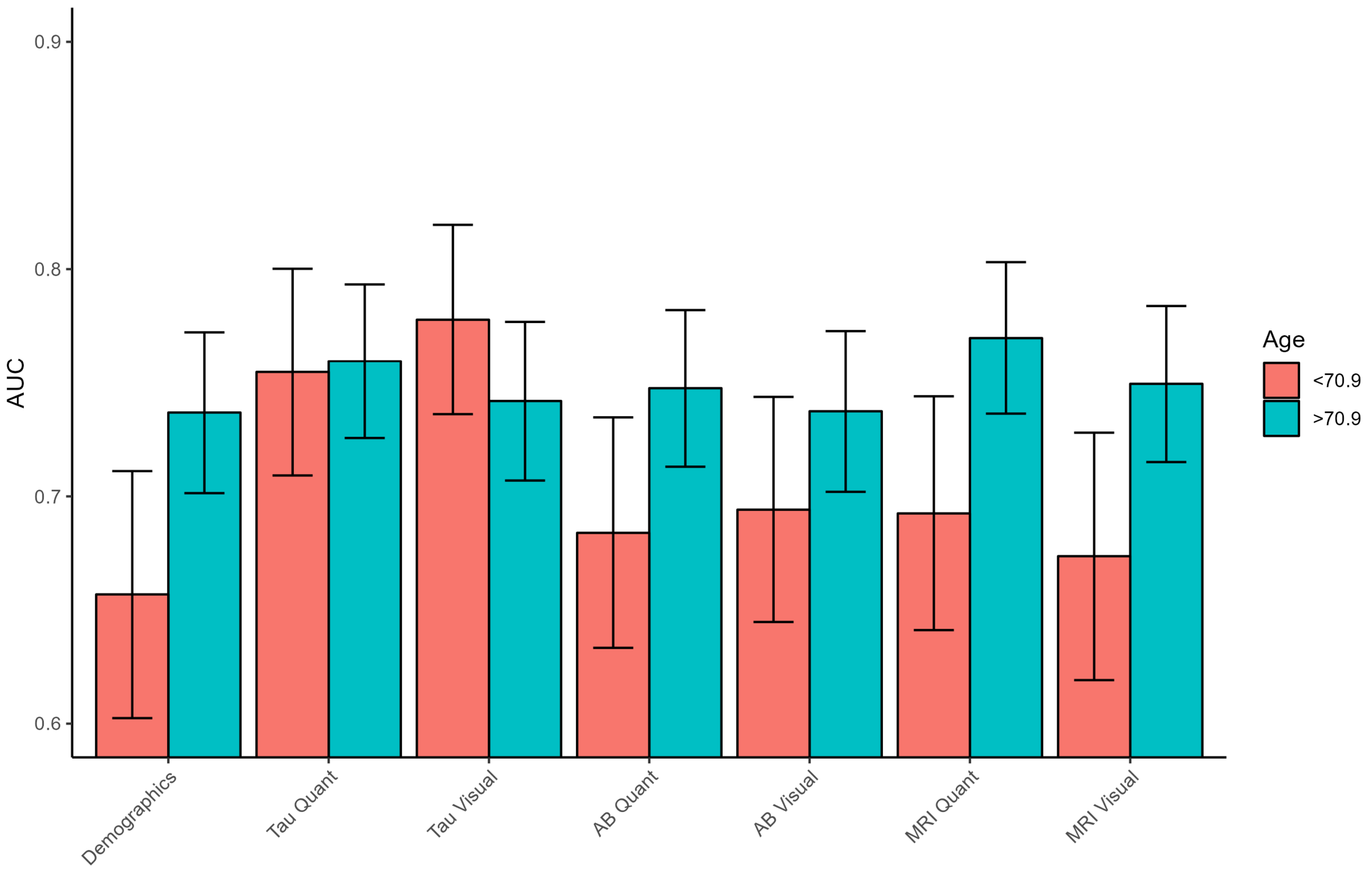

Validation cohort

© 2024 Groot C et al. *JAMA Neurology*.

All-cause dementia

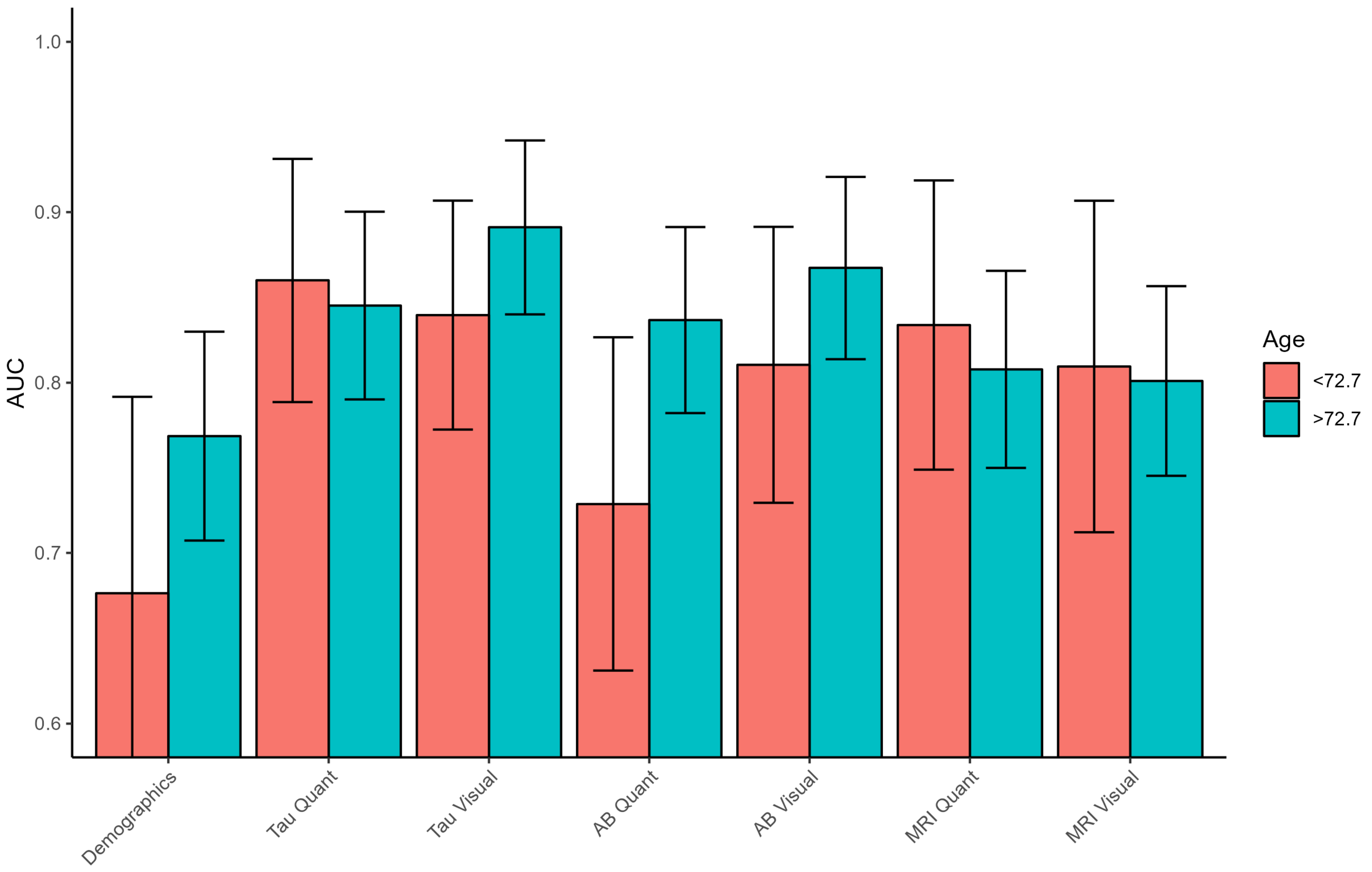

AD dementia

© 2024 Groot C et al. *JAMA Neurology*.

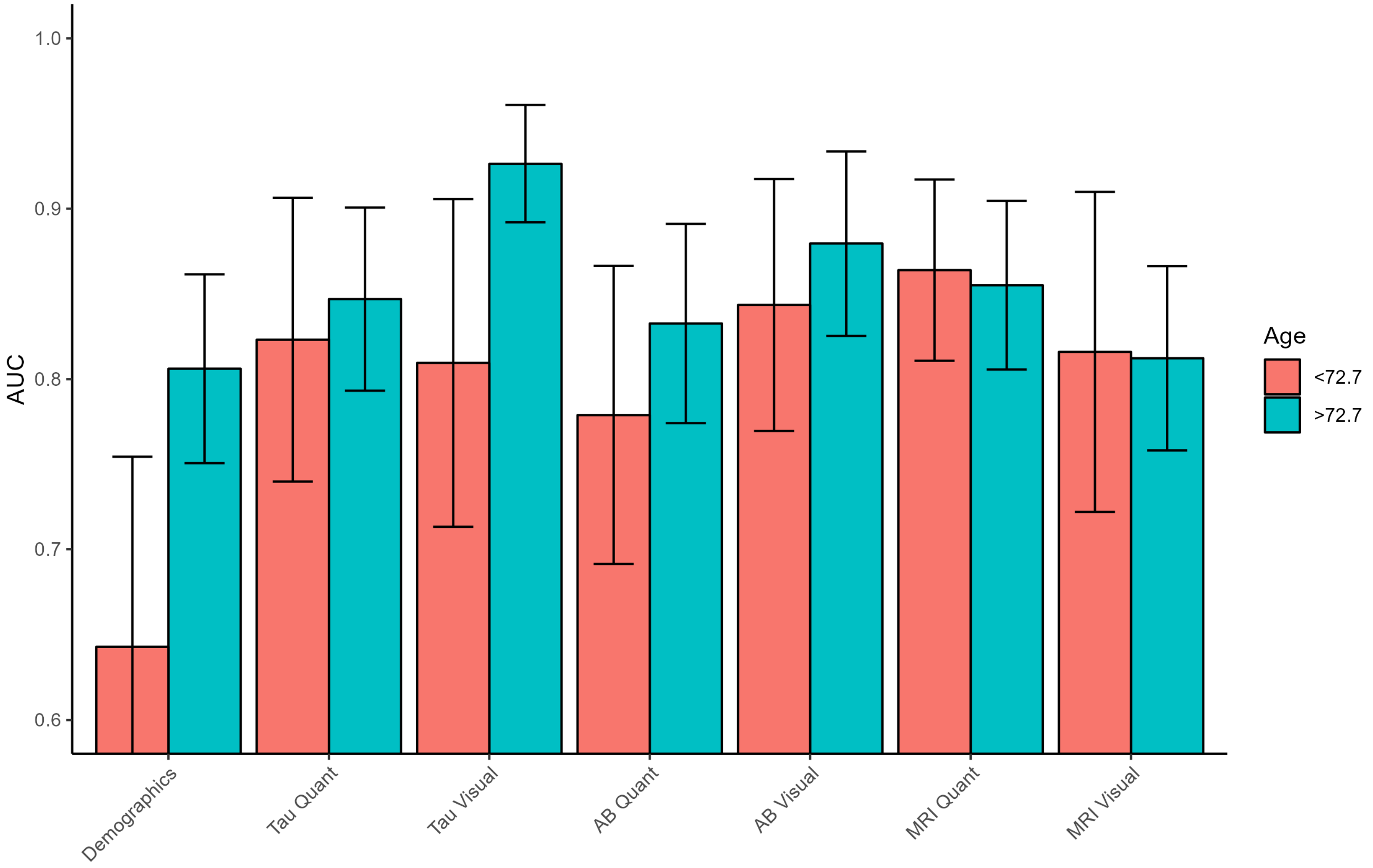

© 2024 Groot C et al. *JAMA Neurology*.

eFigure 4. ROC analyses to distinguish stable MCI from progressors, stratified according to age groups at the mean

We examined the effects of age on the prognostic performance of tau-PET, Aβ-PET and MRI by stratifying our sample at 71 years (the mean in the total sample) and running the ROC analyses assessing the performance of all neuroimaging markers to predict progression to dementia in individuals with MCI. The figure shows bar graphs of the resulting AUC statistics and highlights that the AUCs for tau-PET to predict all-cause and AD dementia were numerically higher in younger individuals than in older individuals, although this difference was not statistically significant when assessed using the DeLong method. Interestingly, the base model was significantly more predictive for conversion to AD dementia in older individuals than in younger individuals. Demographics included age, sex, education, MMSE. The models for neuroimaging markers also included the demographics and all models were additionally corrected for cohort and follow-up time. AUC–area under the curve, AB - amyloid-beta.

© 2024 Groot C et al. *JAMA Neurology.*

Discovery cohort

All-cause dementia

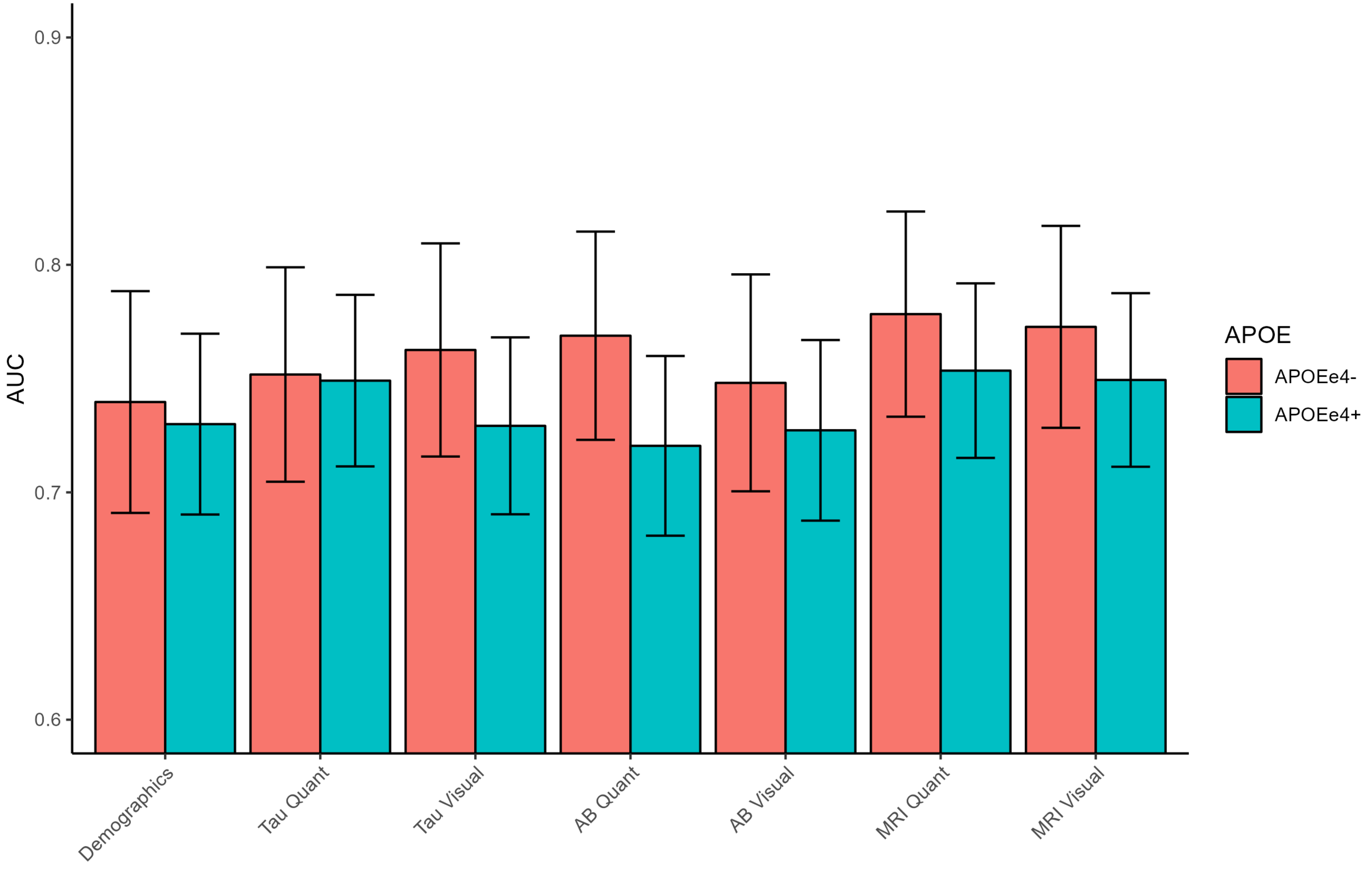

© 2024 Groot C et al. *JAMA Neurology*.

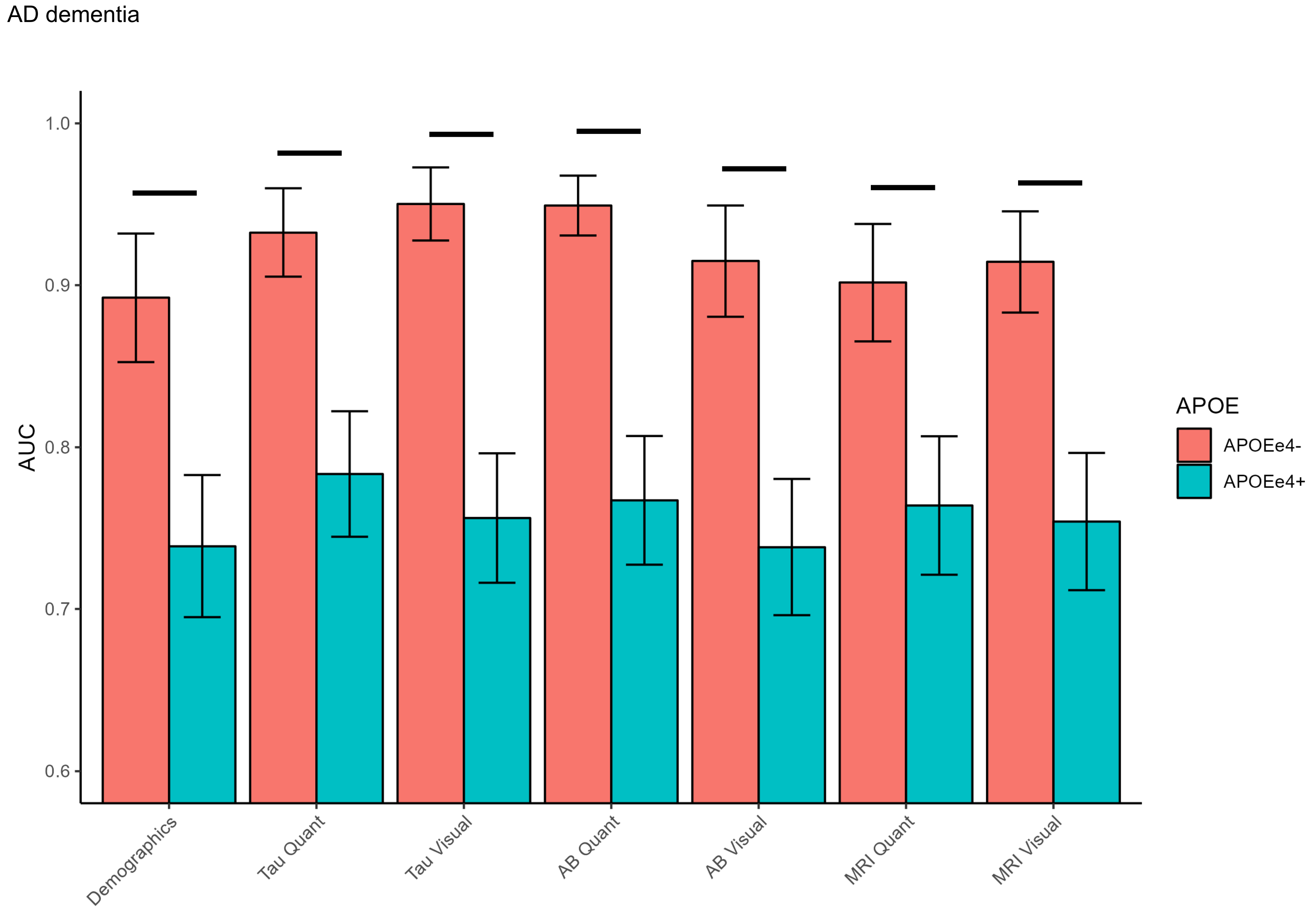

© 2024 Groot C et al. *JAMA Neurology*.

Validation cohort

All-cause dementia

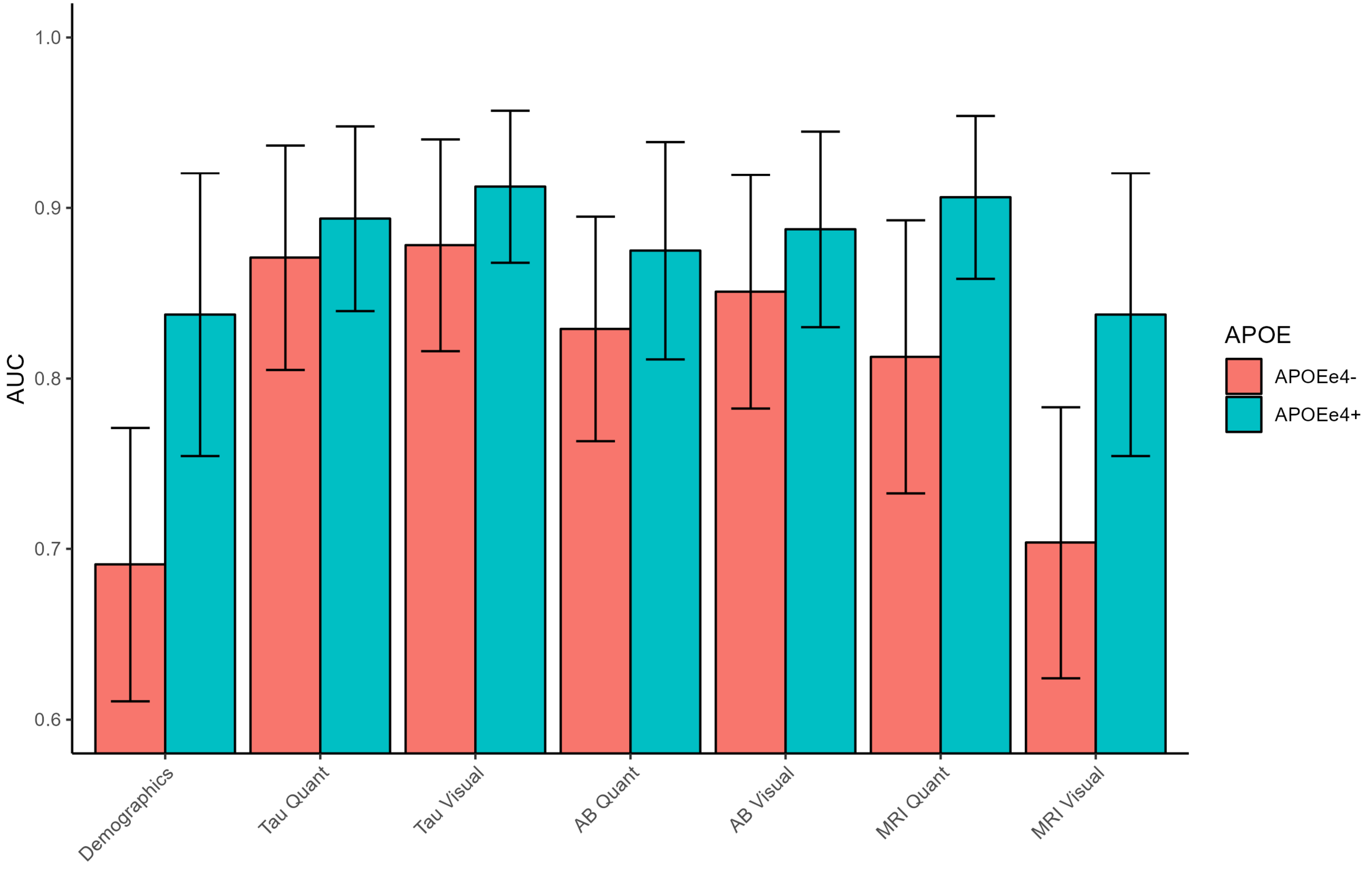

© 2024 Groot C et al. *JAMA Neurology*.

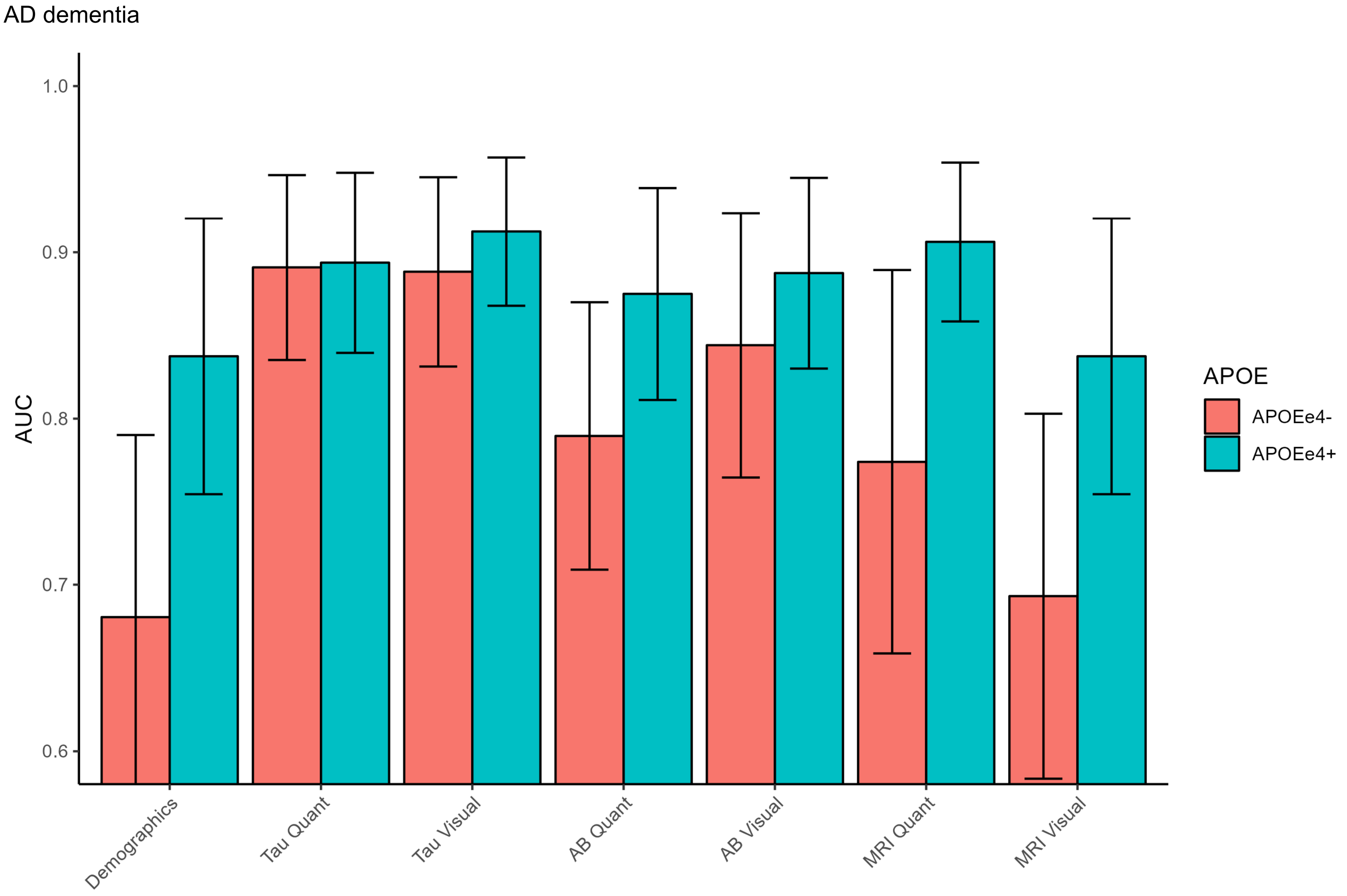

© 2024 Groot C et al. *JAMA Neurology*.

eFigure 5. ROC analyses to distinguish stable MCI from progressors, stratified according to APOEε4 genotype

To assess the effects of APOEε4 carriership on the performance of our neuroimaging markers, we stratified the sample according to APOEε4 carriership and ran the ROC analyses. The AUCs of tau-PET and Aβ-PET to predict AD dementia are higher in APOEε4-negative individuals than in APOEε4-positive individuals, and this difference was significant for the tau-PET visual reads (p=0.01) and AD-signature cortical thickness (p=0.02). Demographics included age, sex, education, MMSE. The models for neuroimaging markers also included the demographics and all models were additionally corrected for cohort and follow-up time. Differences in AUCs were assessed using the DeLong method. * = significantly different compared to the other APOE group, AUC–area under the curve, AB - amyloid-beta.

© 2024 Groot C et al. *JAMA Neurology.*

Discovery cohort

All-cause dementia

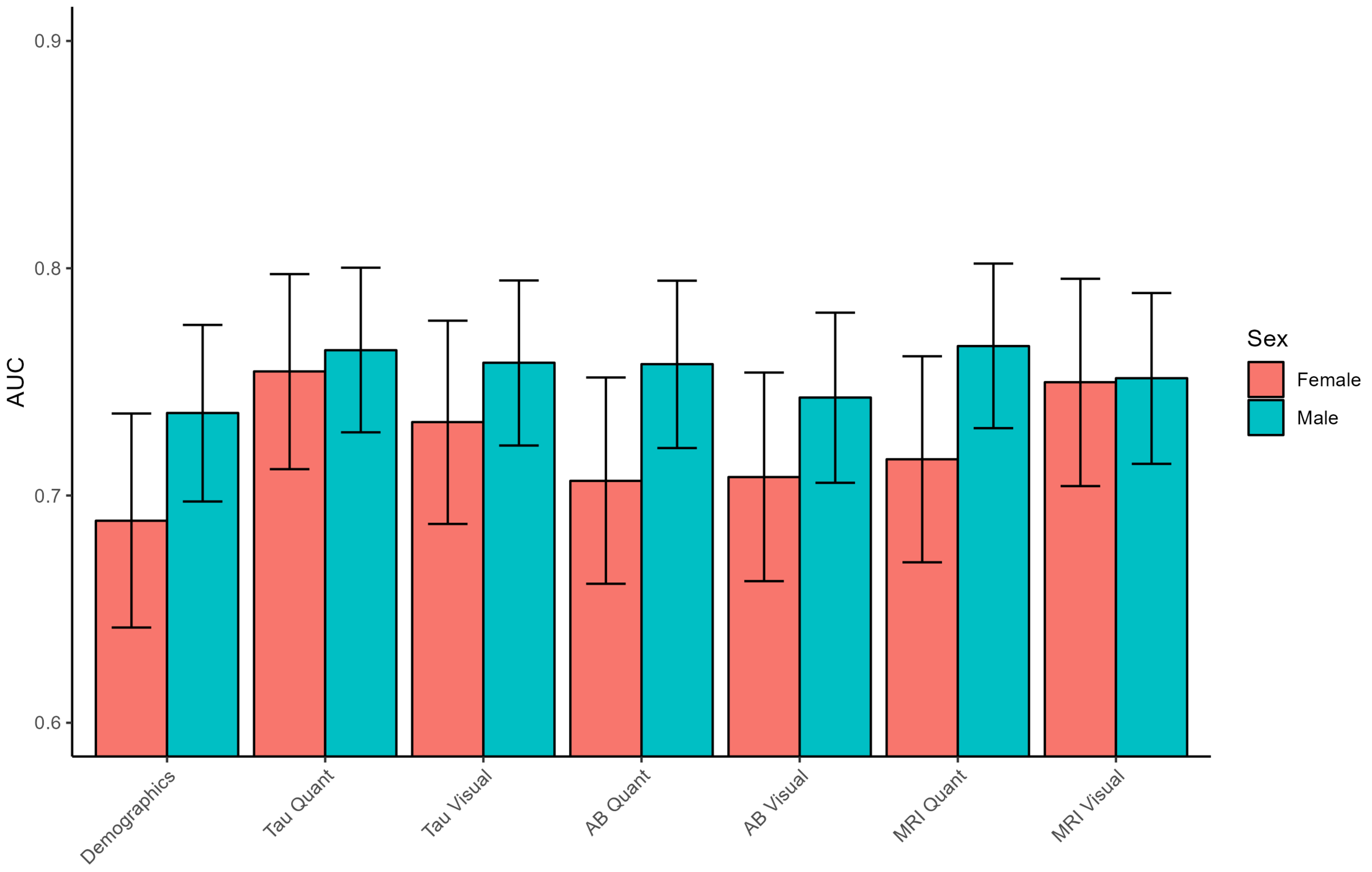

© 2024 Groot C et al. *JAMA Neurology.*

AD dementia

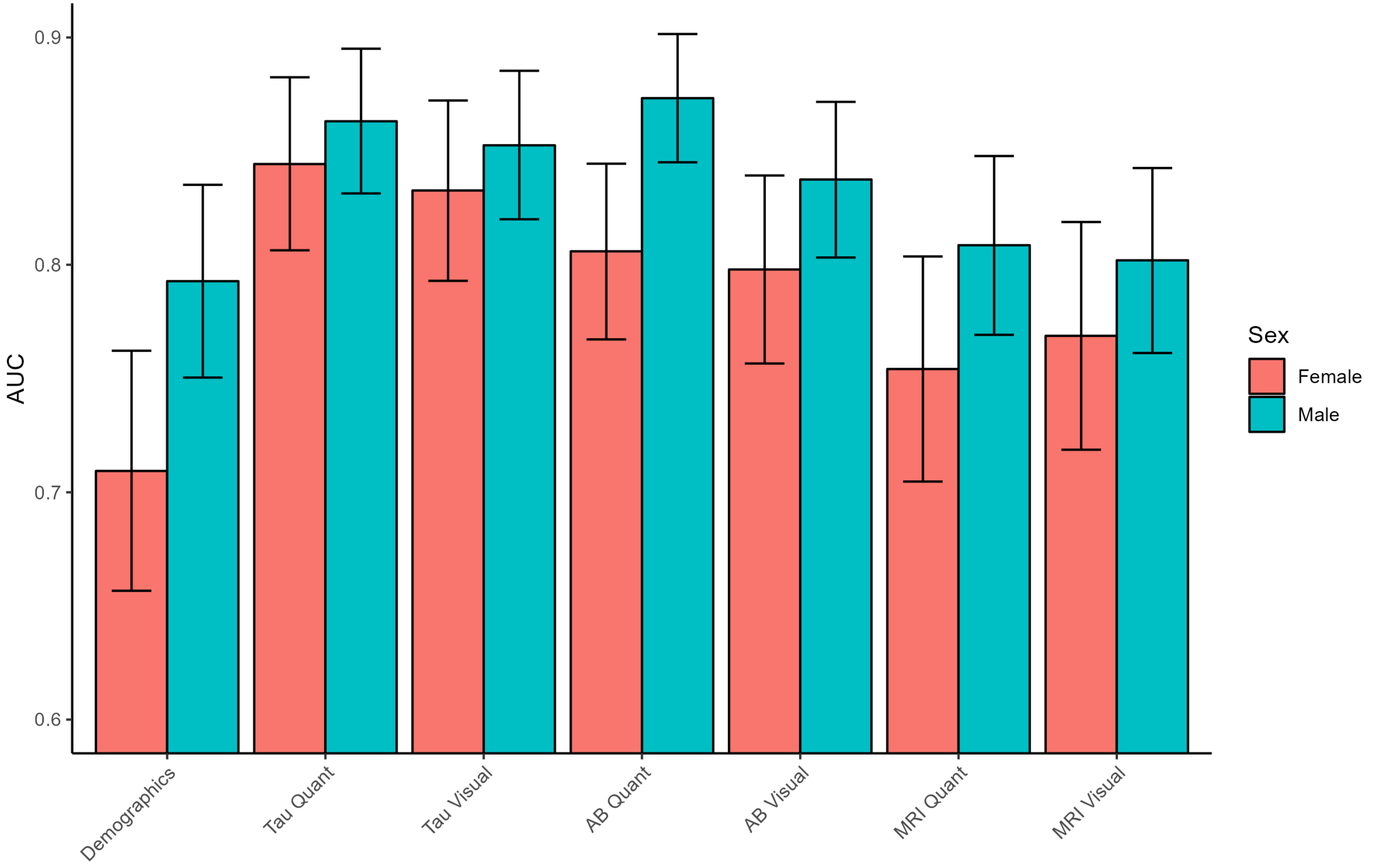

© 2024 Groot C et al. *JAMA Neurology*.

Validation cohort

All-cause dementia

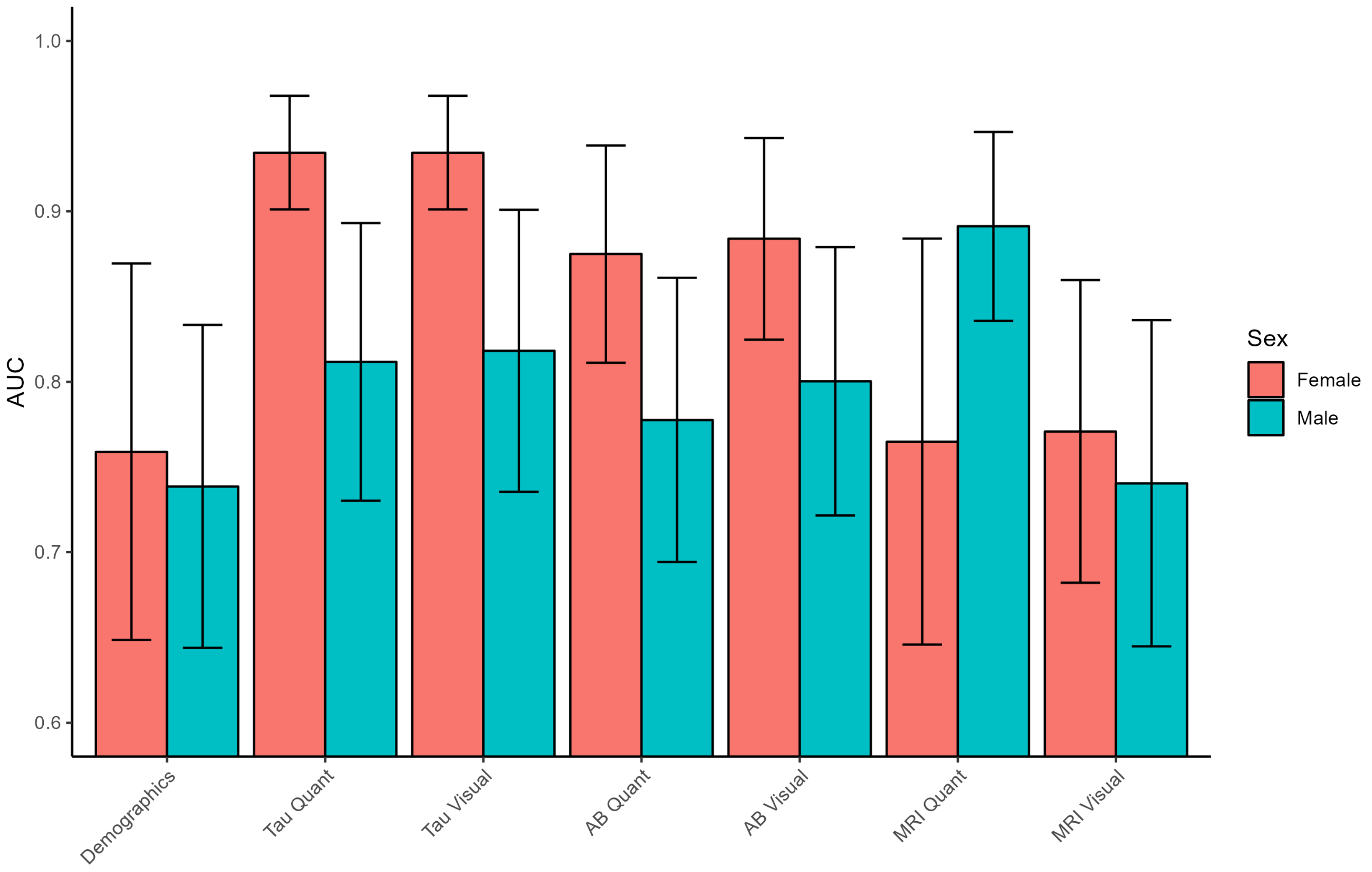

© 2024 Groot C et al. *JAMA Neurology*.

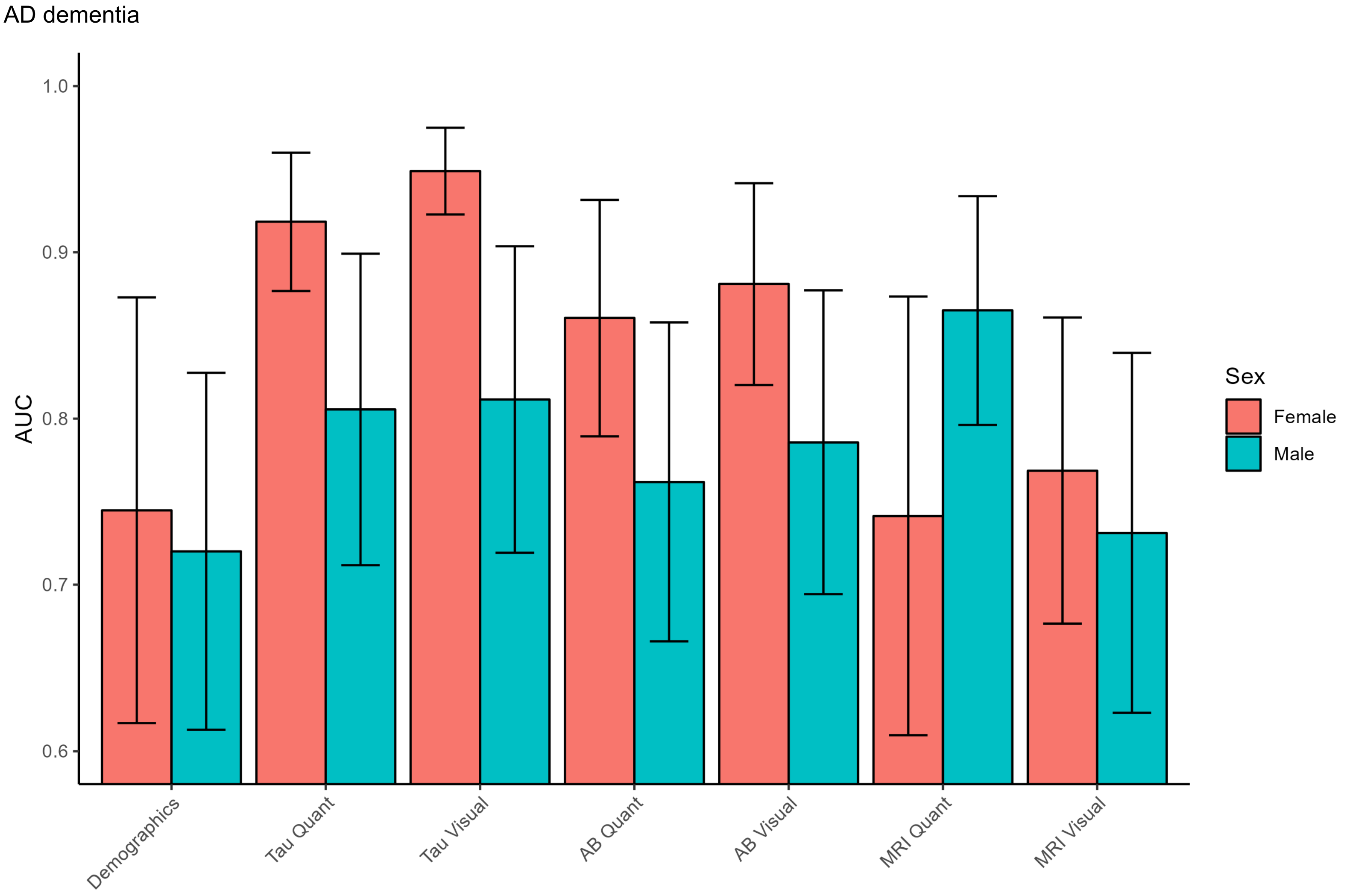

© 2024 Groot C et al. *JAMA Neurology*.

eFigure 6. ROC analyses to distinguish stable MCI from progressors, stratified according to sex

To assess the effects of sex on the performance of our neuroimaging markers, we stratified the sample according to sex and ran the ROC analyses. The AUCs of the tau-PET measures, especially when predicting AD dementia, were numerically higher in females than in males, although this difference was not statistically significant when assessed using the DeLong method. Also, the AUCs of the AD signature cortical thickness measures to predict all-cause and AD dementia are higher in males than in female, but this difference was not statistically significant using the DeLong method. Demographics included age, sex, education, MMSE. The models for neuroimaging markers also included the demographics and all models were additionally corrected for cohort and follow-up time. Differences in AUCs were assessed using the DeLong method. * = significantly different between the sexes (there were no differences), AUC–area under the curve, AB - amyloid-beta

© 2024 Groot C et al. *JAMA Neurology.*

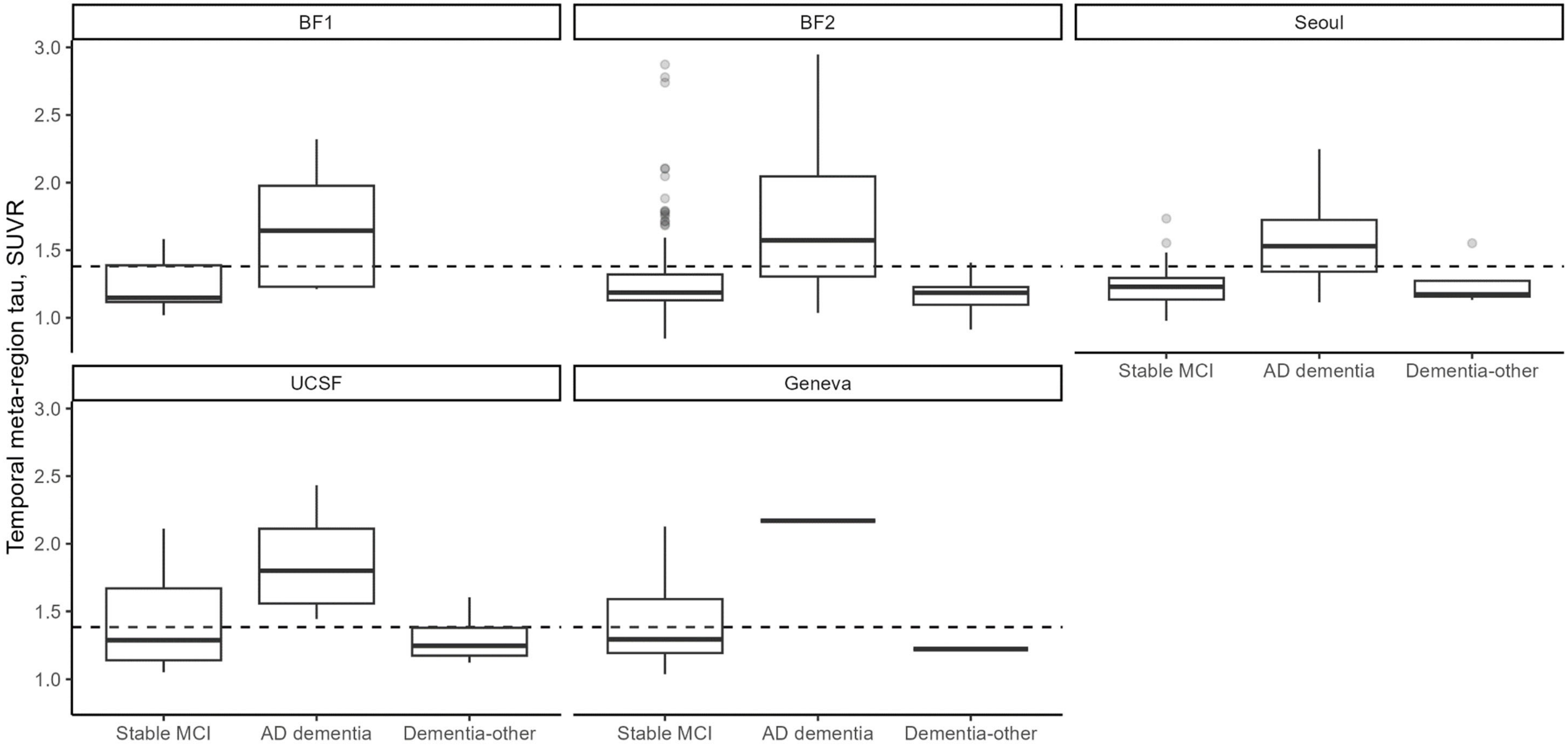

© 2024 Groot C et al. *JAMA Neurology*.

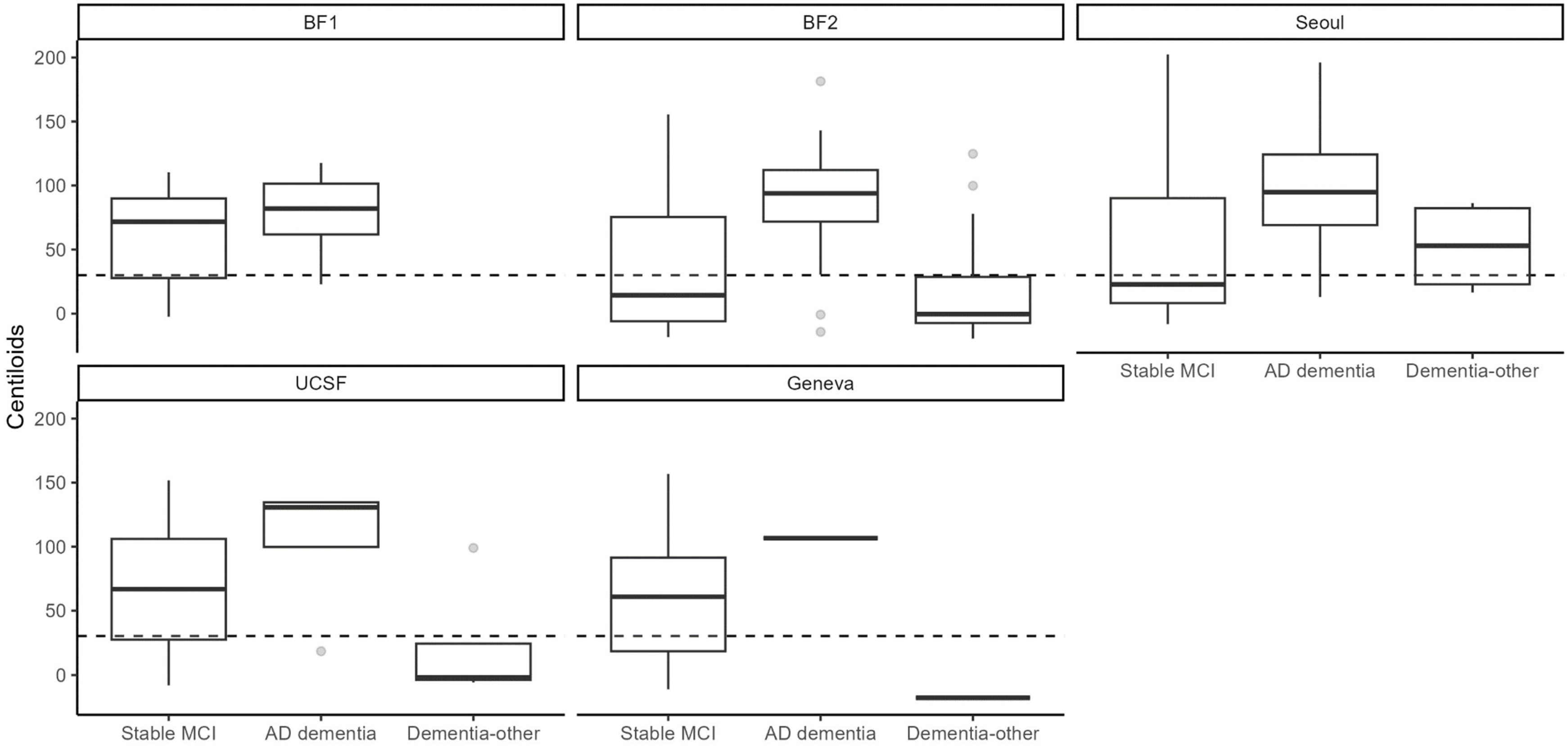

© 2024 Groot C et al. *JAMA Neurology*.

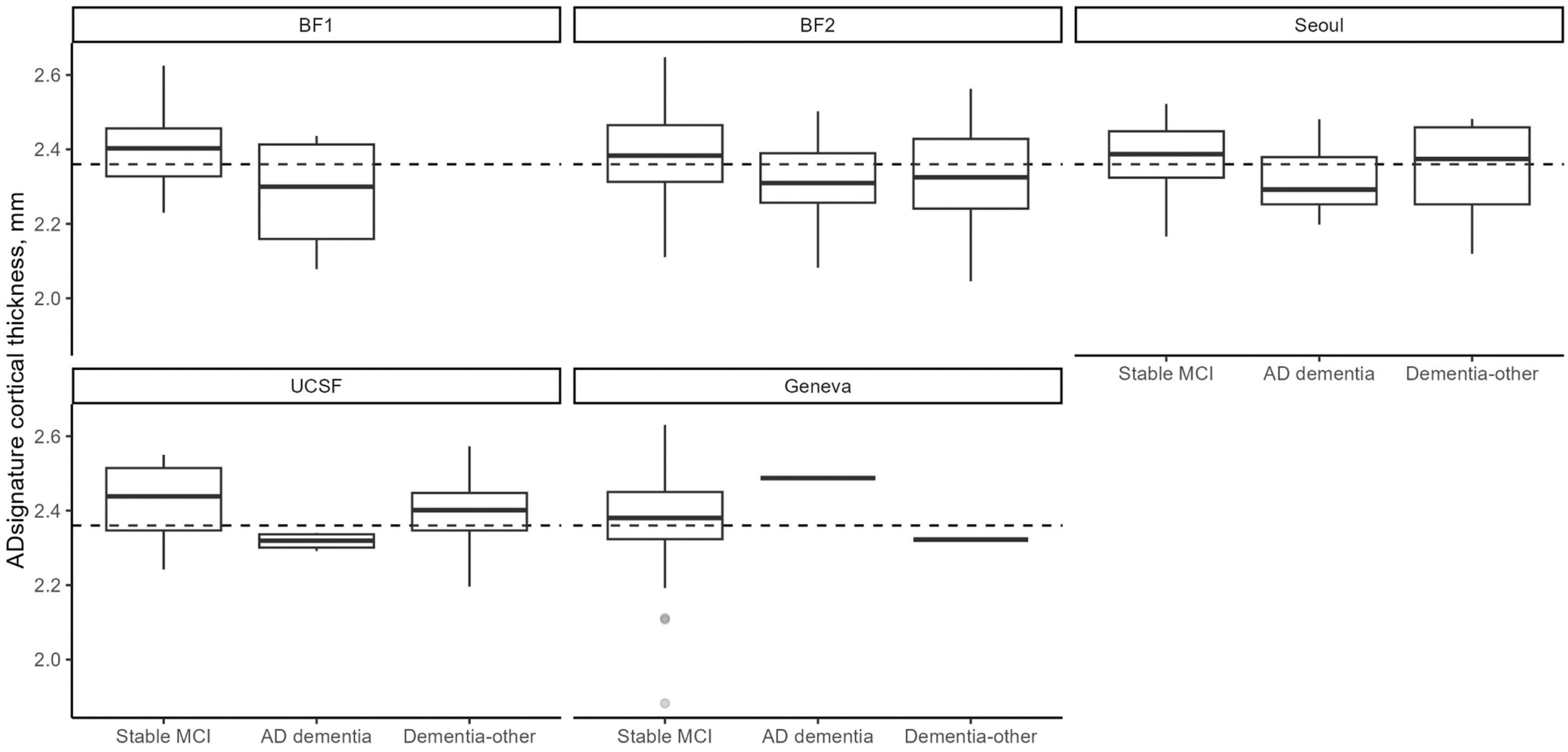

eFigure 7. Data distribution of primary neuroimaging markers across cohorts in the discovery cohort

In order to examine whether cohort differences could impact our results, we have plotted the distribution of our primary neuroimaging measure according to stable MCI and progressors groups and stratified per cohort. The data distribution was comparable across cohorts. AD–Alzheimer's disease, AB–amyloid-β, SUVR–standardized uptake value ratio, mm–millimeter

© 2024 Groot C et al. *JAMA Neurology.*

References

1. Leuzy A, Smith R, Ossenkoppele R, *et al*. Diagnostic Performance of RO948 F 18 Tau Positron Emission Tomography in the Differentiation of Alzheimer Disease From Other Neurodegenerative Disorders. *JAMA Neurol*. Aug 1 2020;77(8):955-965. doi:10.1001/jamaneurol.2020.0989
2. Palmqvist S, Janelidze S, Quiroz YT, *et al*. Discriminative Accuracy of Plasma Phospho-tau217 for Alzheimer Disease vs Other Neurodegenerative Disorders. *Jama*. Aug 25 2020;324(8):772-781. doi:10.1001/jama.2020.12134
3. Smith R, Schöll M, Leuzy A, *et al*. Head-to-head comparison of tau positron emission tomography tracers [18 F]flortaucipir and [18 F]RO948. *European journal of nuclear medicine and molecular imaging*. 2020;47(2):342-354. doi:10.1007/S00259-019-04496-0
4. Klunk WE, Koeppe RA, Price JC, *et al*. The Centiloid Project: standardizing quantitative amyloid plaque estimation by PET. *Alzheimers Dement*. Jan 2015;11(1):1-15.e1-4. doi:10.1016/j.jalz.2014.07.003
5. Petersen RC, Smith GE, Waring SC, Ivnik RJ, Tangalos EG, Kokmen E. Mild cognitive impairment: clinical characterization and outcome. *Archives of neurology*. 1999;56(3):303-8.
6. Albert MS, DeKosky ST, Dickson D, *et al*. The diagnosis of mild cognitive impairment due to Alzheimer's disease: Recommendations from the National Institute on Aging-Alzheimer's Association workgroups on diagnostic guidelines for Alzheimer's disease. *Alzheimer's and Dementia*. 2011;7(3):270-279. doi:10.1016/j.jalz.2011.03.008
7. Leuzy A, Janelidze S, Mattsson-Carlgren N, *et al*. Comparing the Clinical Utility and Diagnostic Performance of Cerebrospinal Fluid P-Tau181, P-Tau217 and P-Tau231 Assays. *Neurology*. Sep 7 2021;doi:10.1212/wnl.0000000000012727
8. Jack CR, Jr., Wiste HJ, Weigand SD, *et al*. Defining imaging biomarker cut points for brain aging and Alzheimer's disease. *Alzheimers Dement*. Mar 2017;13(3):205-216. doi:10.1016/j.jalz.2016.08.005
9. Dickerson BC, Bakkour A, Salat DH, *et al*. The cortical signature of Alzheimer's disease: Regionally specific cortical thinning relates to symptom severity in very mild to mild AD dementia and is detectable in asymptomatic amyloid-positive individuals. *Cerebral Cortex*. 2009;19(3):497-510. doi:10.1093/cercor/bhn113

© 2024 Groot C et al. *JAMA Neurology.*
