# Supplementary material for: Tau Positron Emission Tomography for Predicting Dementia in Individuals With Mild Cognitive Impairment
Source: JAMA Neurol. 2024 Jun 10;81(8):845–56. doi: 10.1001/jamaneurol.2024.1612 (PMC11165418; doi:10.1001/jamaneurol.2024.1612)
Supplement: Supplement 2. — Data sharing statement [file jamaneurol-e241612-s002.pdf]

Data Sharing Statement

Groot. Tau Positron Emission Tomography for Predicting Dementia in Individuals With Mild Cognitive Impairment. *JAMA Neurol*. Published June 10, 2024.
doi:10.1001/jamaneurol.2024.1612

Data
Data available: No

Additional Information
Explanation for why data not available: Anonymized data may be shared upon request from a qualified academic investigator for the sole purpose of replicating procedures and results presented in the article and as long as data transfer is in agreement with European Union legislation on the general data protection regulation and decisions by the Swedish Ethical Review Authority and Region Skåne, which should be regulated in a material transfer agreement.
